# Supplementary figures and images for: BZR1 promotes pluripotency acquisition and callus development through direct regulation of ARF7 and ARF19
Source: EMBO Rep. 2025 Jun 26;26(14):3554–73. doi: 10.1038/s44319-025-00433-5 (PMC12287390; doi:10.1038/s44319-025-00433-5)

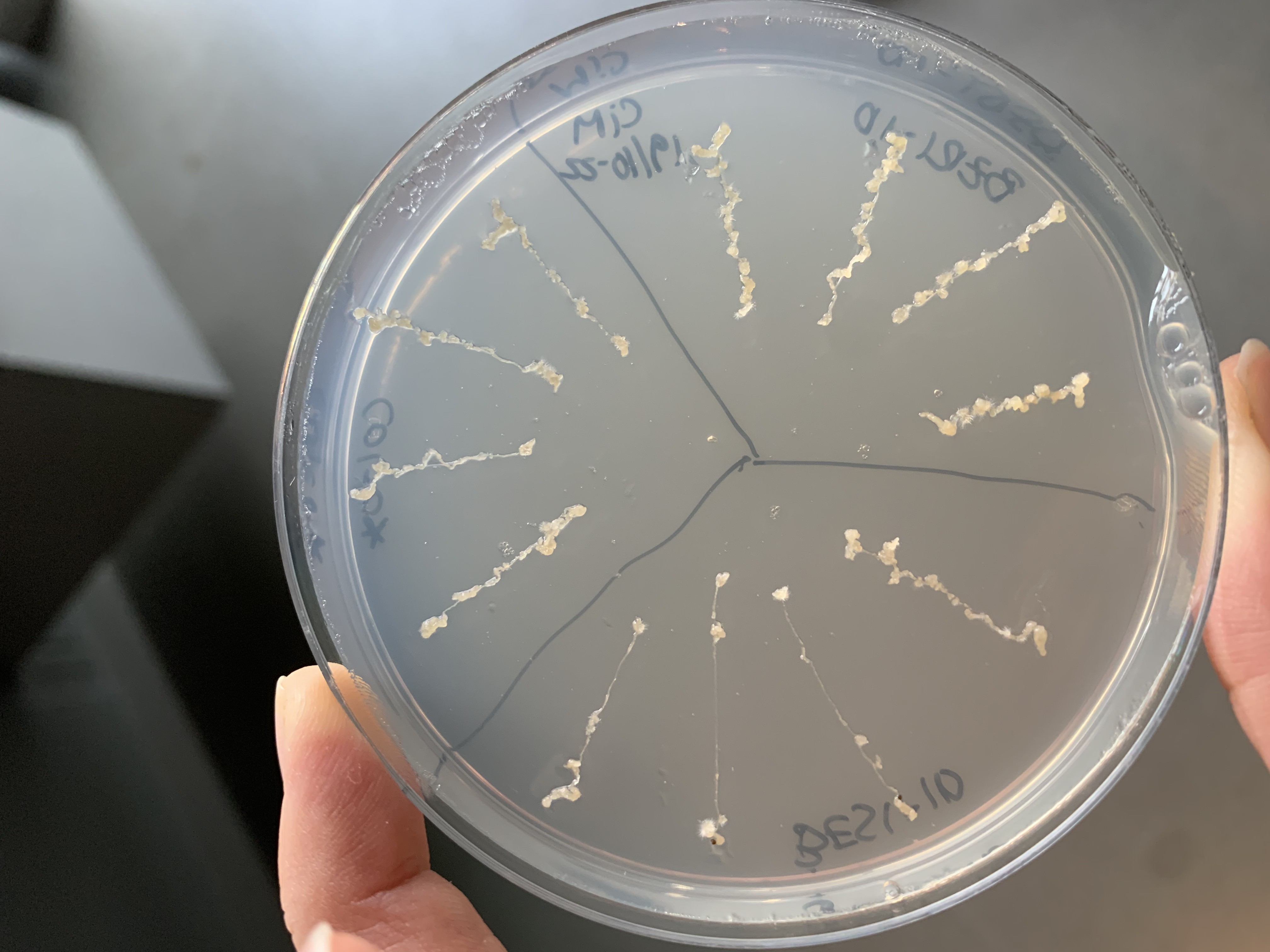

Supplement: Supplementary file 2 — Source data Fig. 1 [file 44319_2025_433_MOESM2_ESM.zip › Fig 1/1A/CIM/IMG_0009.jpg]

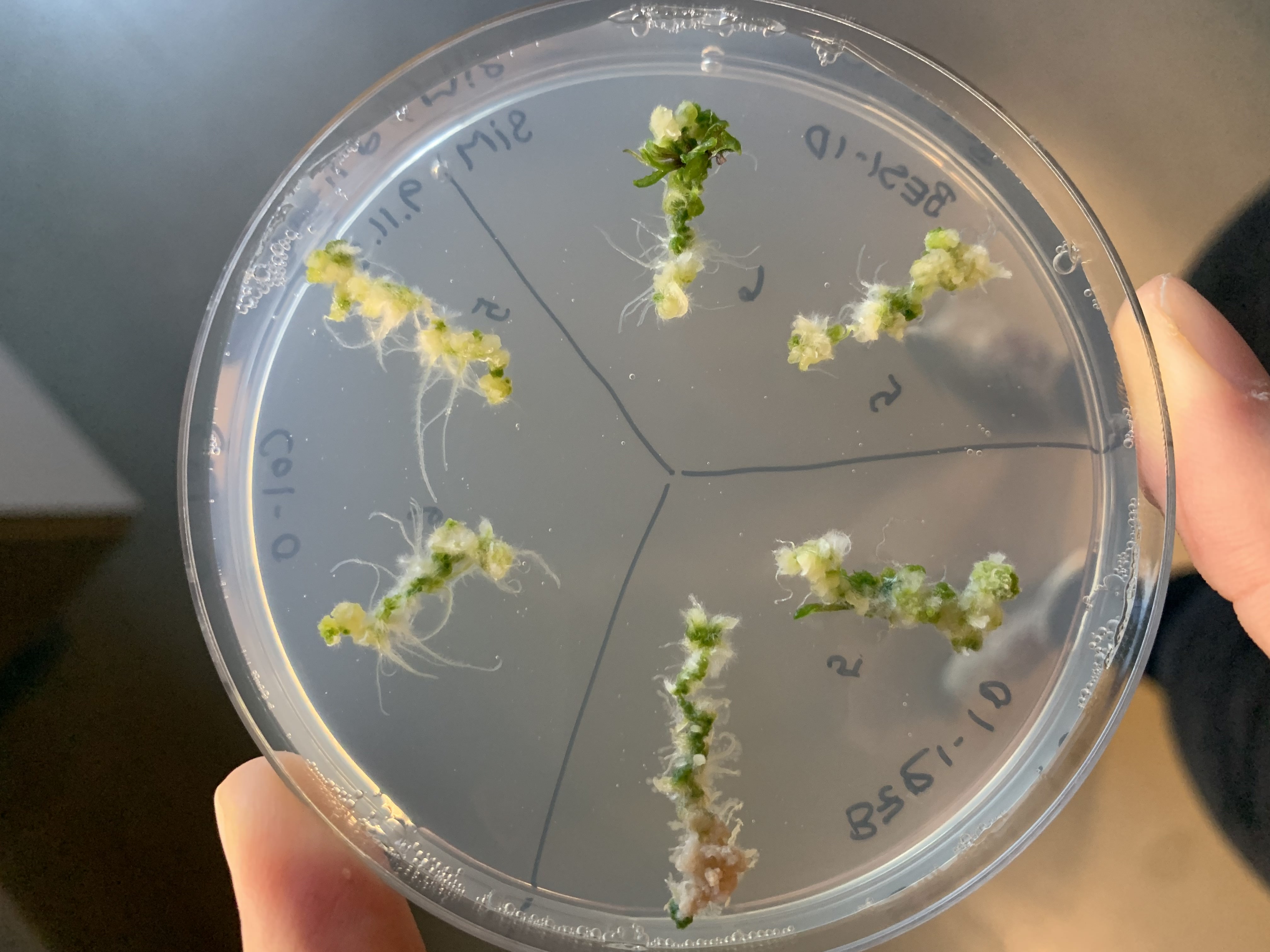

Supplement: Supplementary file 2 — Source data Fig. 1 [file 44319_2025_433_MOESM2_ESM.zip › Fig 1/1A/SIM/bes1-D_IMG_0126.jpg]

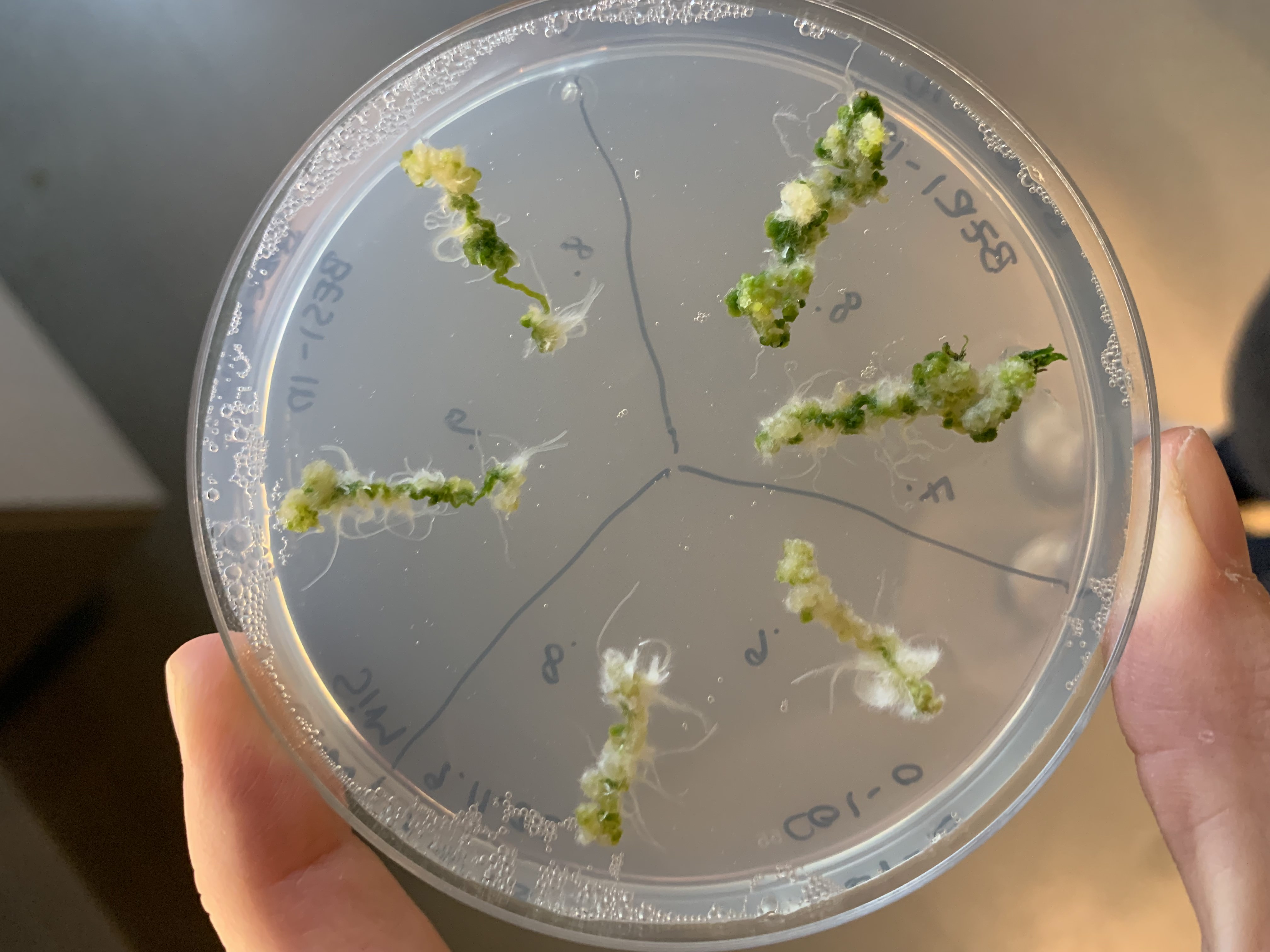

Supplement: Supplementary file 2 — Source data Fig. 1 [file 44319_2025_433_MOESM2_ESM.zip › Fig 1/1A/SIM/bzr1-D_IMG_0128.jpg]

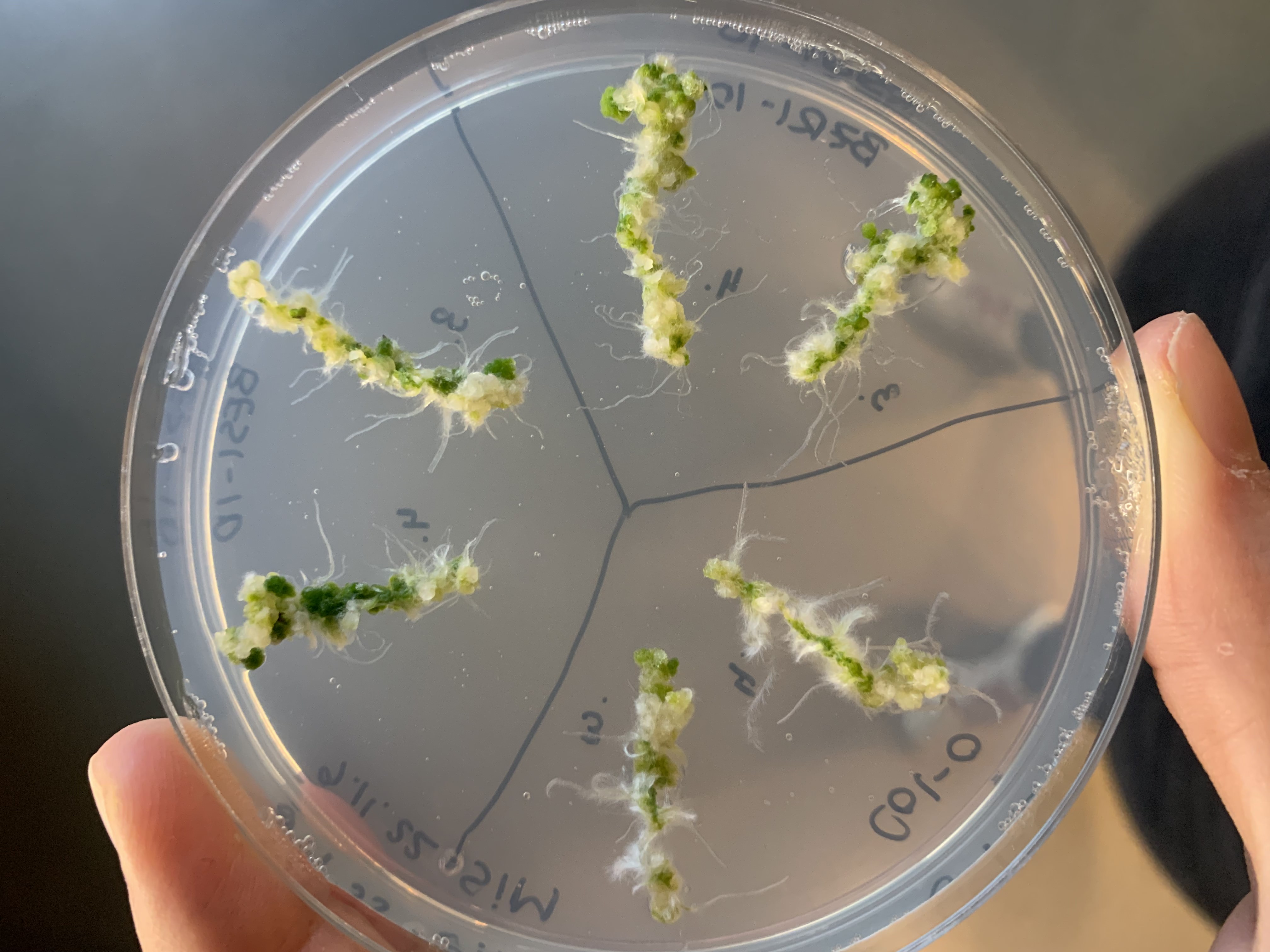

Supplement: Supplementary file 2 — Source data Fig. 1 [file 44319_2025_433_MOESM2_ESM.zip › Fig 1/1A/SIM/Col-0_IMG_0125.jpg]

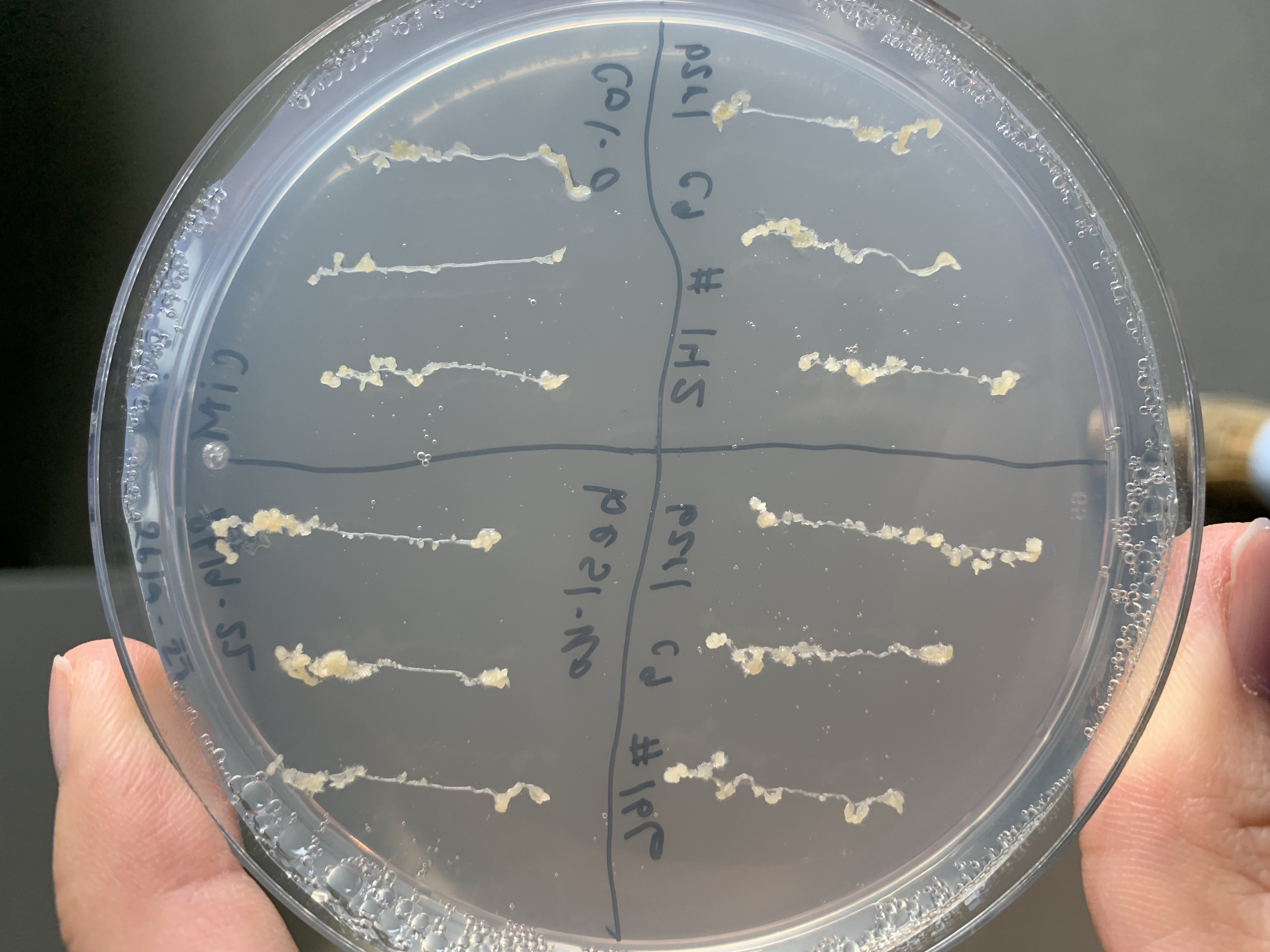

Supplement: Supplementary file 3 — Source data Fig. 2 [file 44319_2025_433_MOESM3_ESM.zip › Fig 2/2A/CIM pics/IMG_9881.jpg]

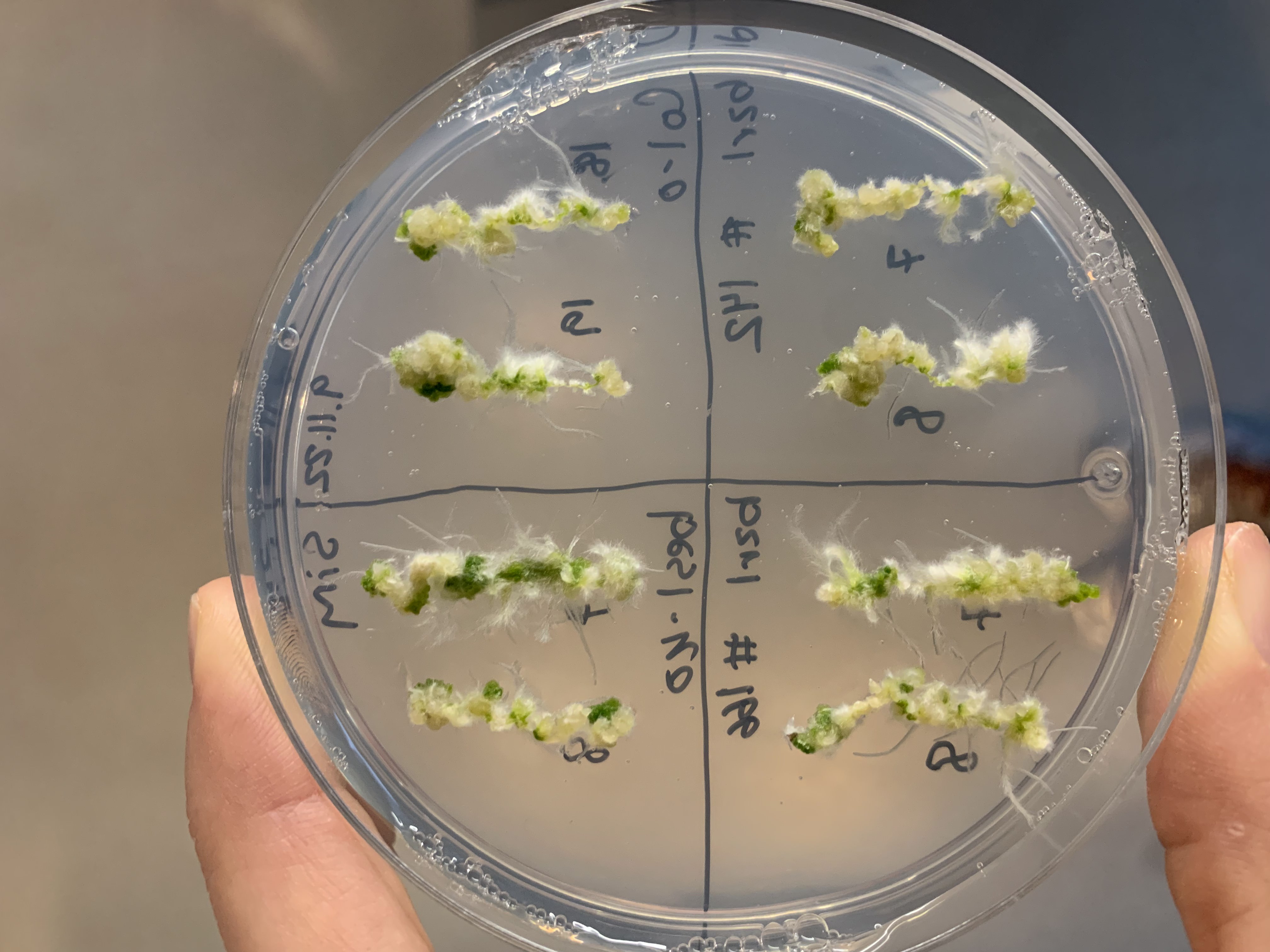

Supplement: Supplementary file 3 — Source data Fig. 2 [file 44319_2025_433_MOESM3_ESM.zip › Fig 2/2A/SIM pics/bes1-ko_IMG_0135.jpg]

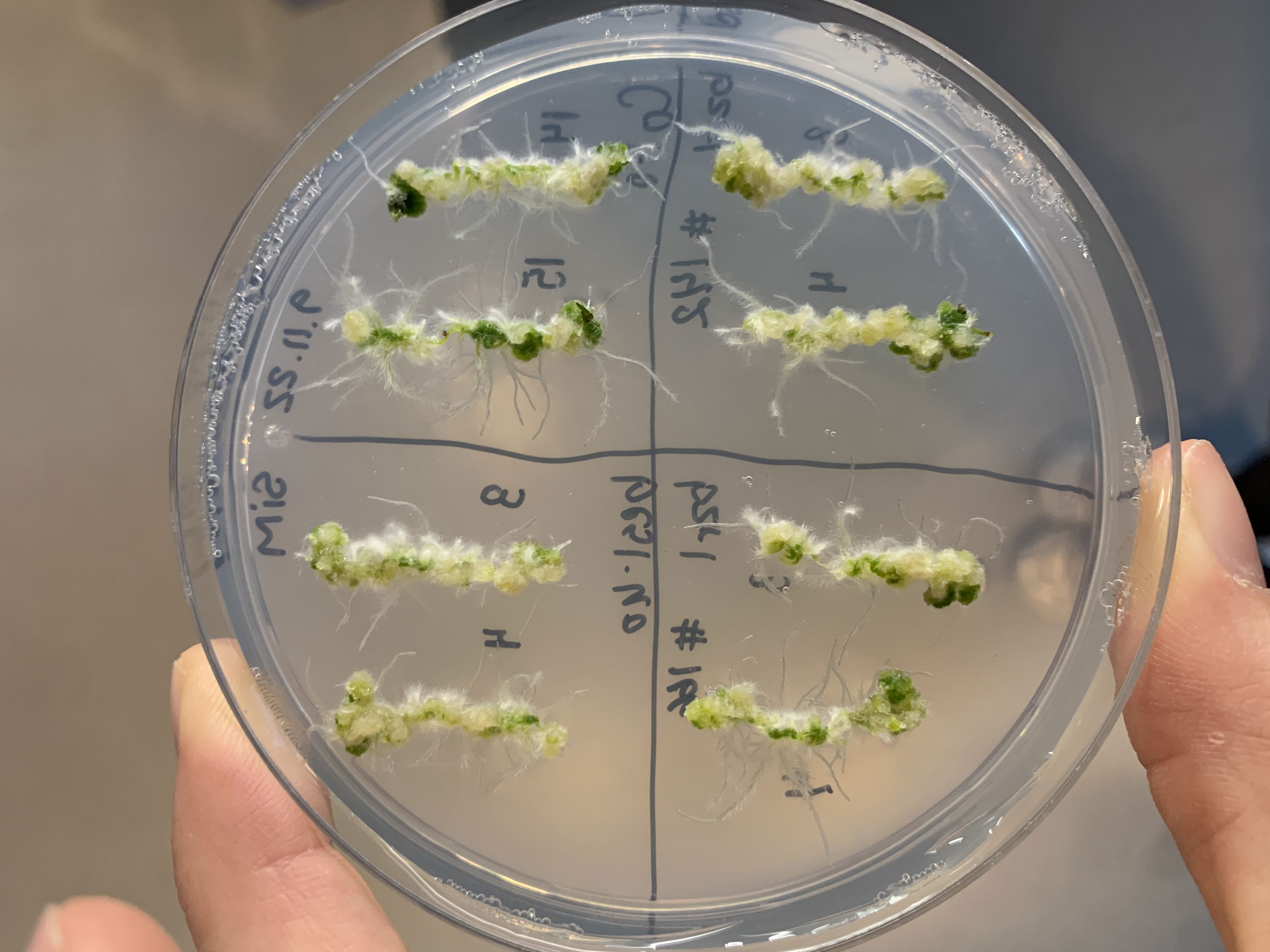

Supplement: Supplementary file 3 — Source data Fig. 2 [file 44319_2025_433_MOESM3_ESM.zip › Fig 2/2A/SIM pics/Col_bzr_c1_c2_IMG_0132.jpg]

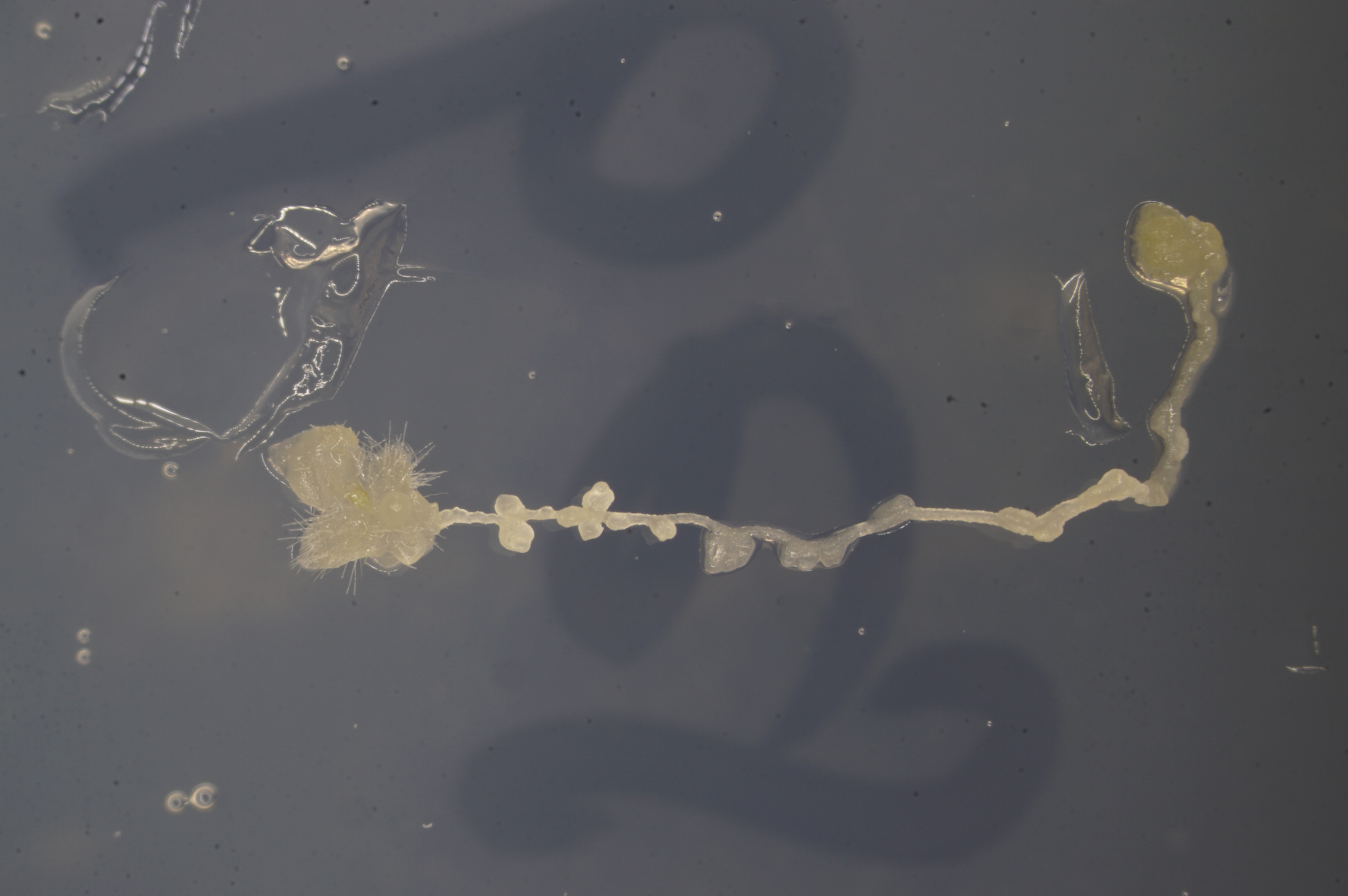

Supplement: Supplementary file 4 — Source data Fig. 3 [file 44319_2025_433_MOESM4_ESM.zip › Fig 3/3A/besD bik1.tif]

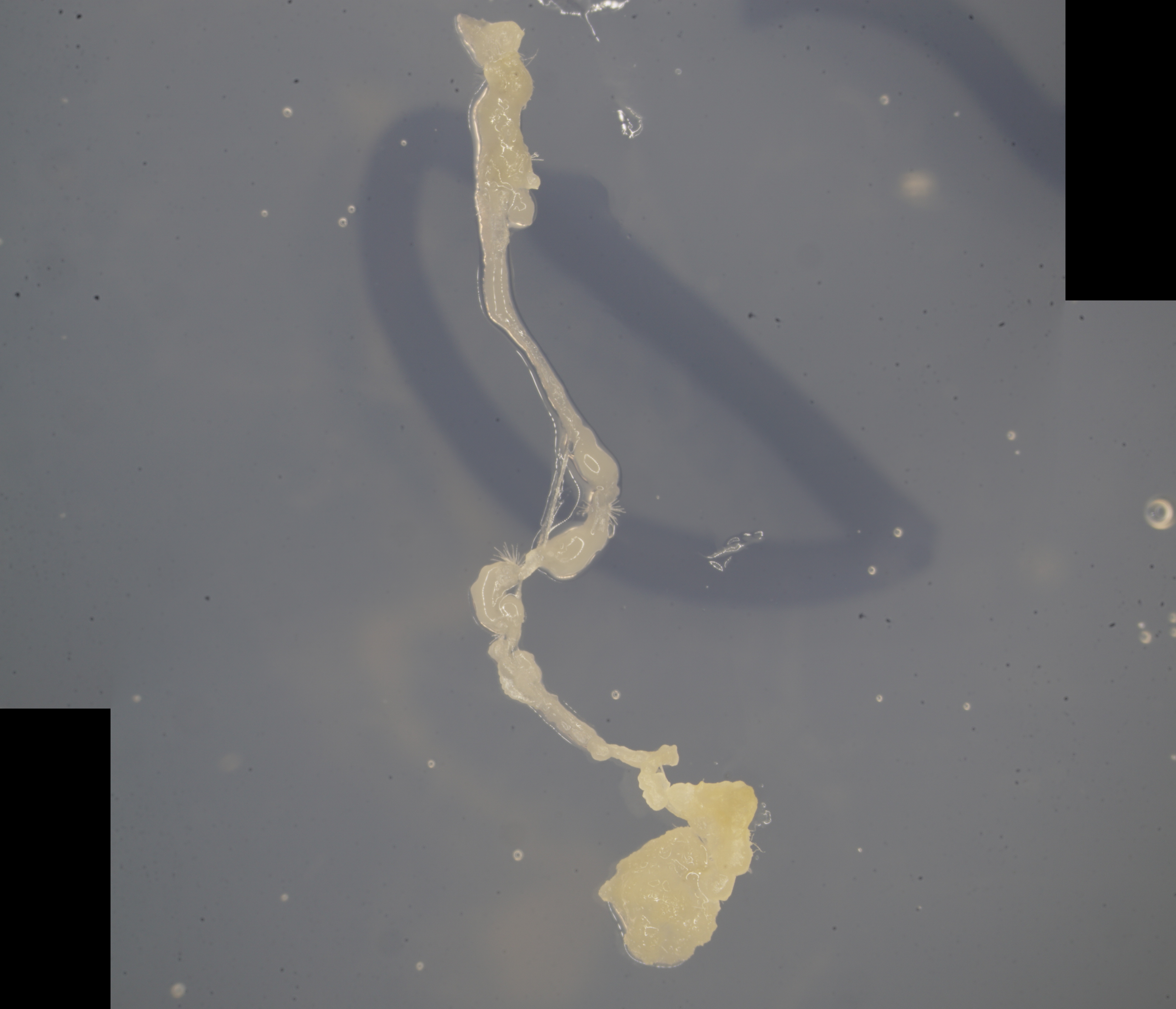

Supplement: Supplementary file 4 — Source data Fig. 3 [file 44319_2025_433_MOESM4_ESM.zip › Fig 3/3A/besD brz2.tif]

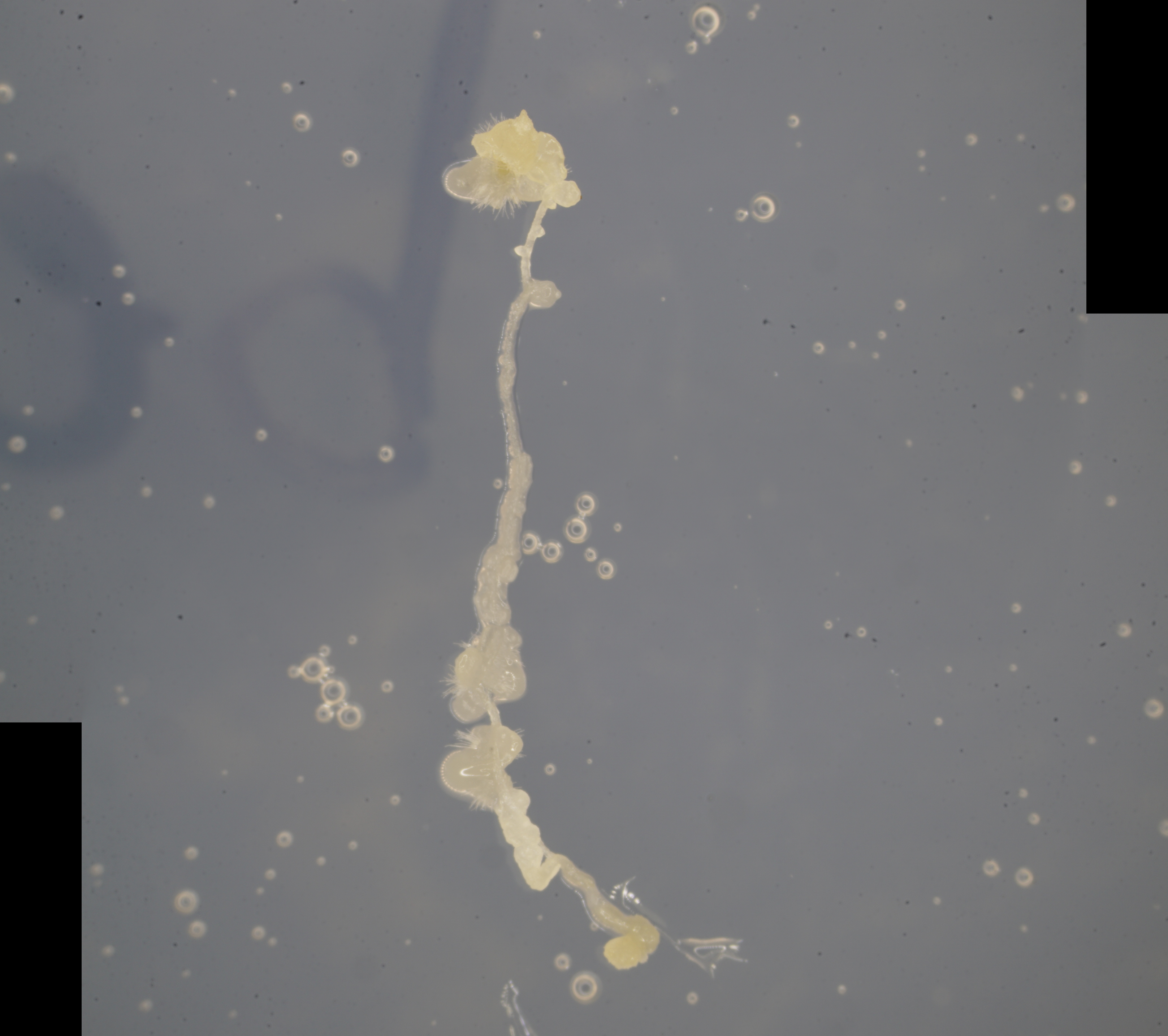

Supplement: Supplementary file 4 — Source data Fig. 3 [file 44319_2025_433_MOESM4_ESM.zip › Fig 3/3A/besD nt1.tif]

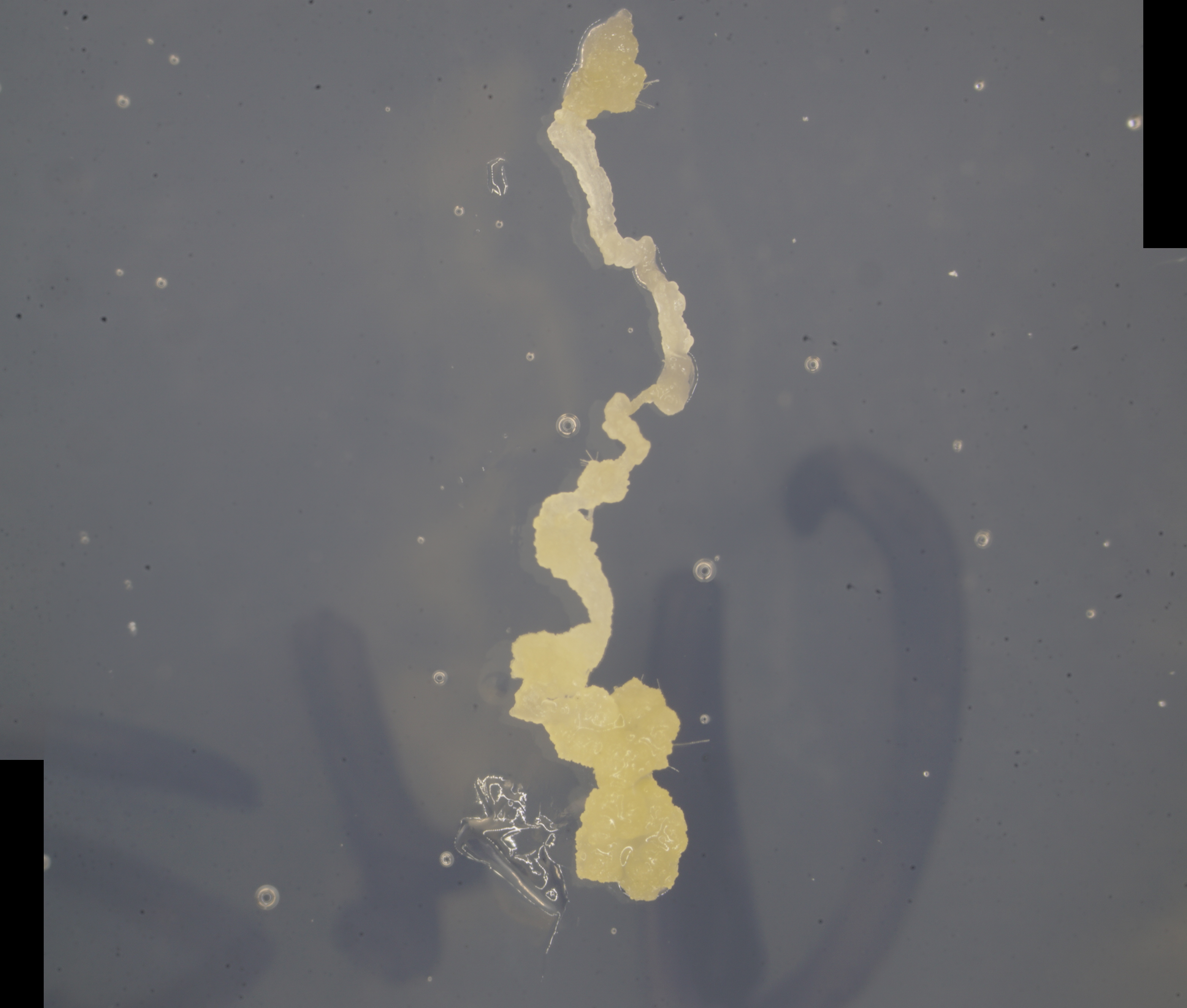

Supplement: Supplementary file 4 — Source data Fig. 3 [file 44319_2025_433_MOESM4_ESM.zip › Fig 3/3A/bzrD bik2.tif]

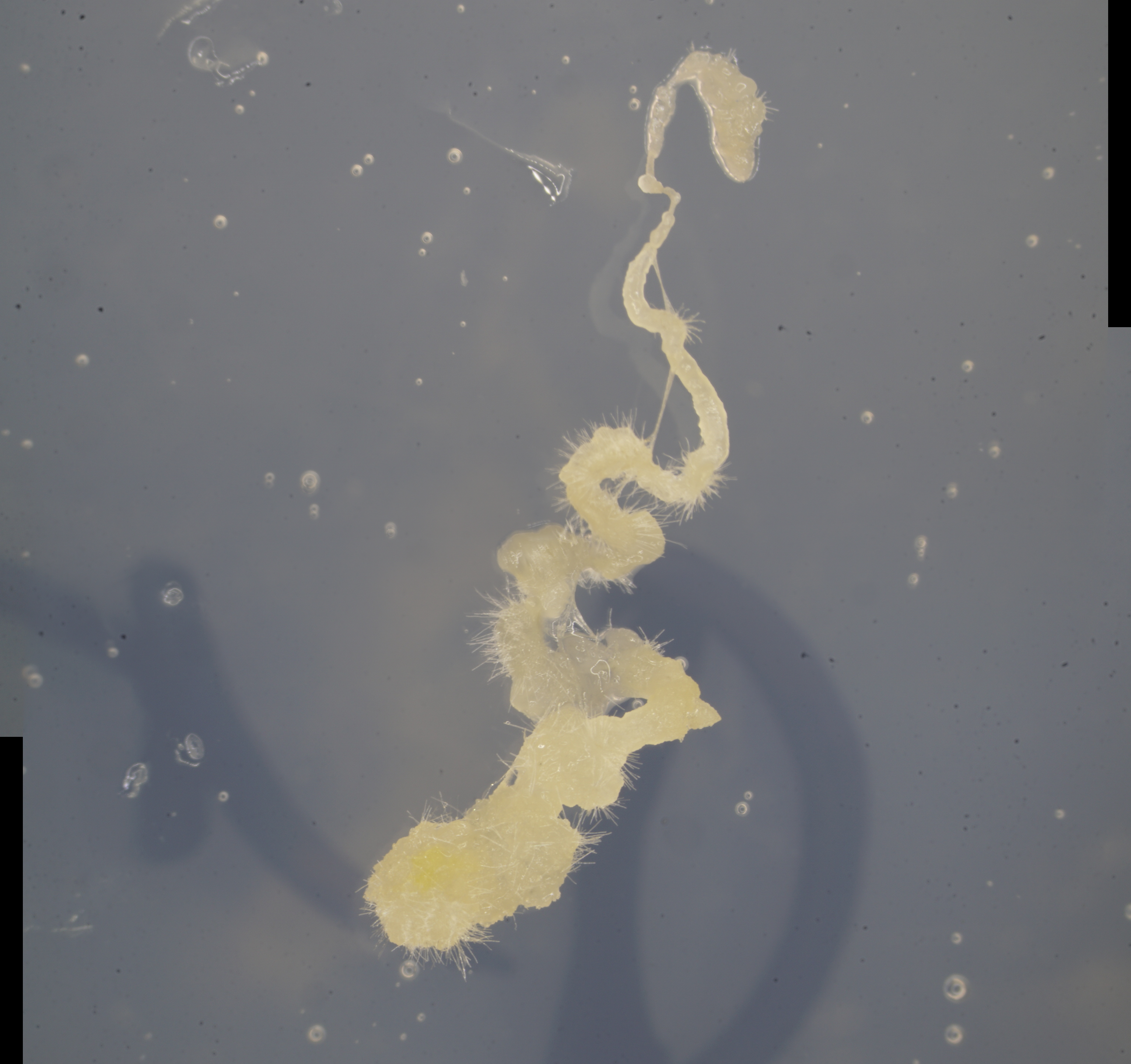

Supplement: Supplementary file 4 — Source data Fig. 3 [file 44319_2025_433_MOESM4_ESM.zip › Fig 3/3A/bzrD brz2.tif]

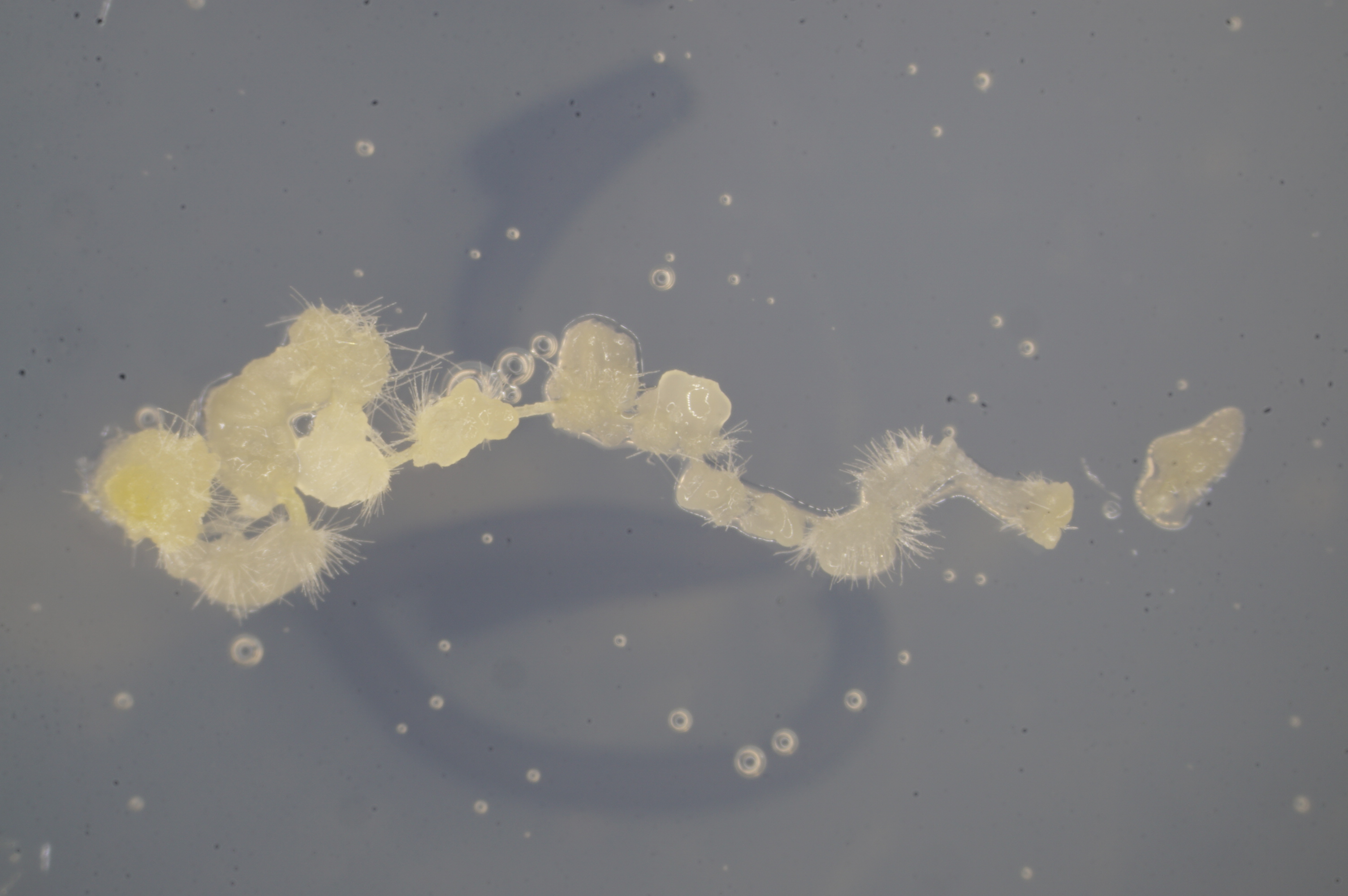

Supplement: Supplementary file 4 — Source data Fig. 3 [file 44319_2025_433_MOESM4_ESM.zip › Fig 3/3A/bzrDnt 2.tif]

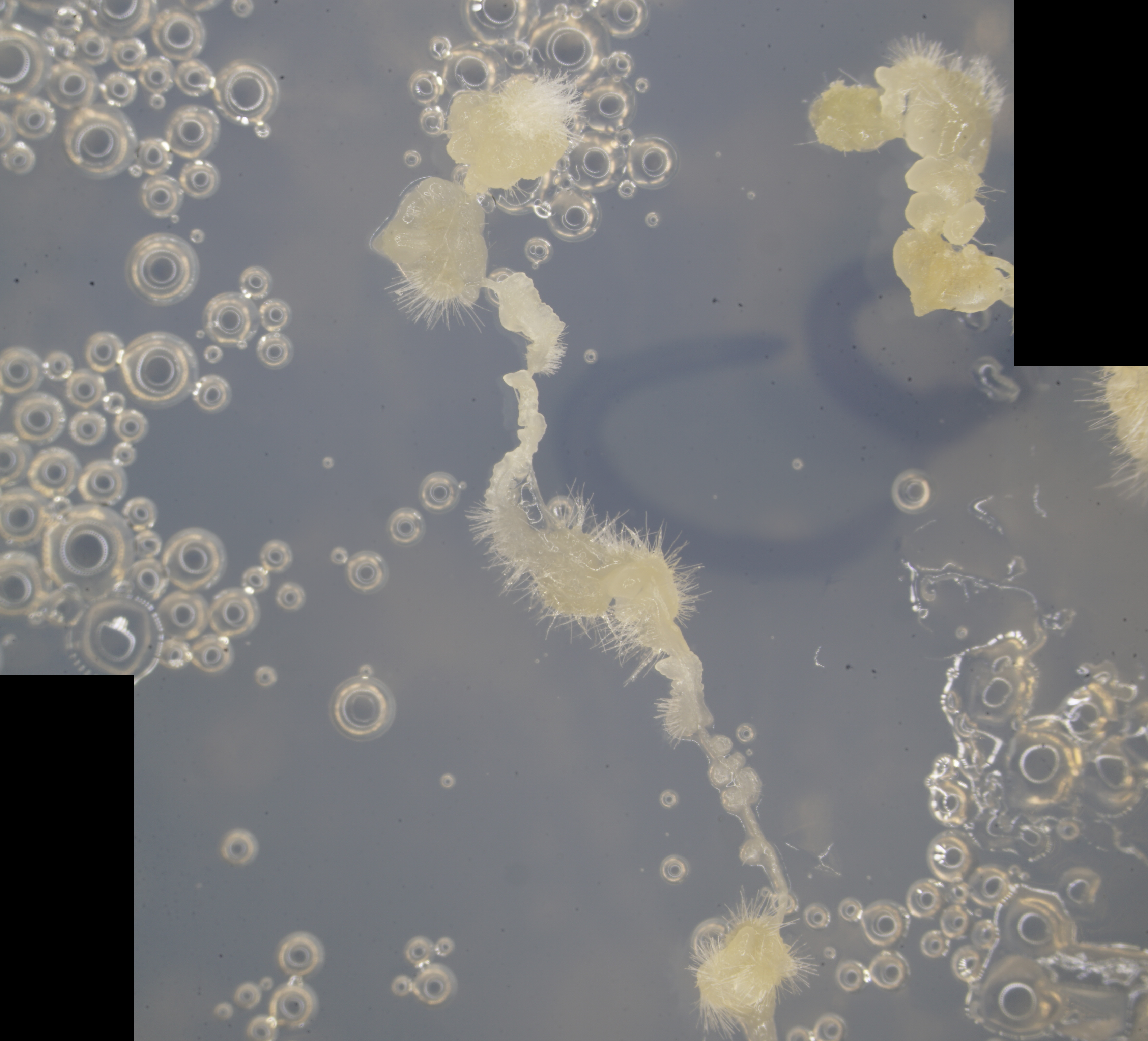

Supplement: Supplementary file 4 — Source data Fig. 3 [file 44319_2025_433_MOESM4_ESM.zip › Fig 3/3A/col 0 nt2.tif]

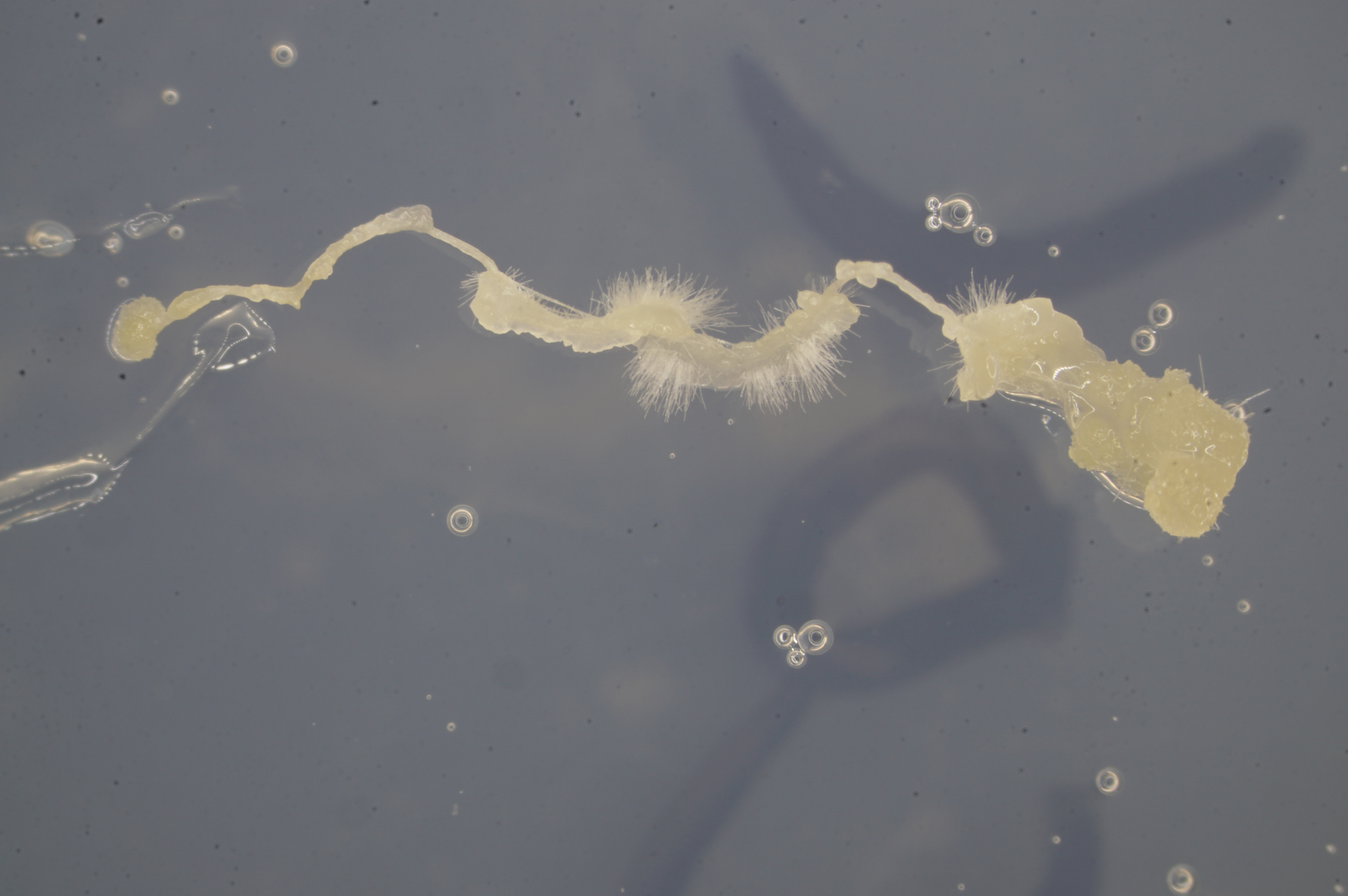

Supplement: Supplementary file 4 — Source data Fig. 3 [file 44319_2025_433_MOESM4_ESM.zip › Fig 3/3A/col bik1.tif]

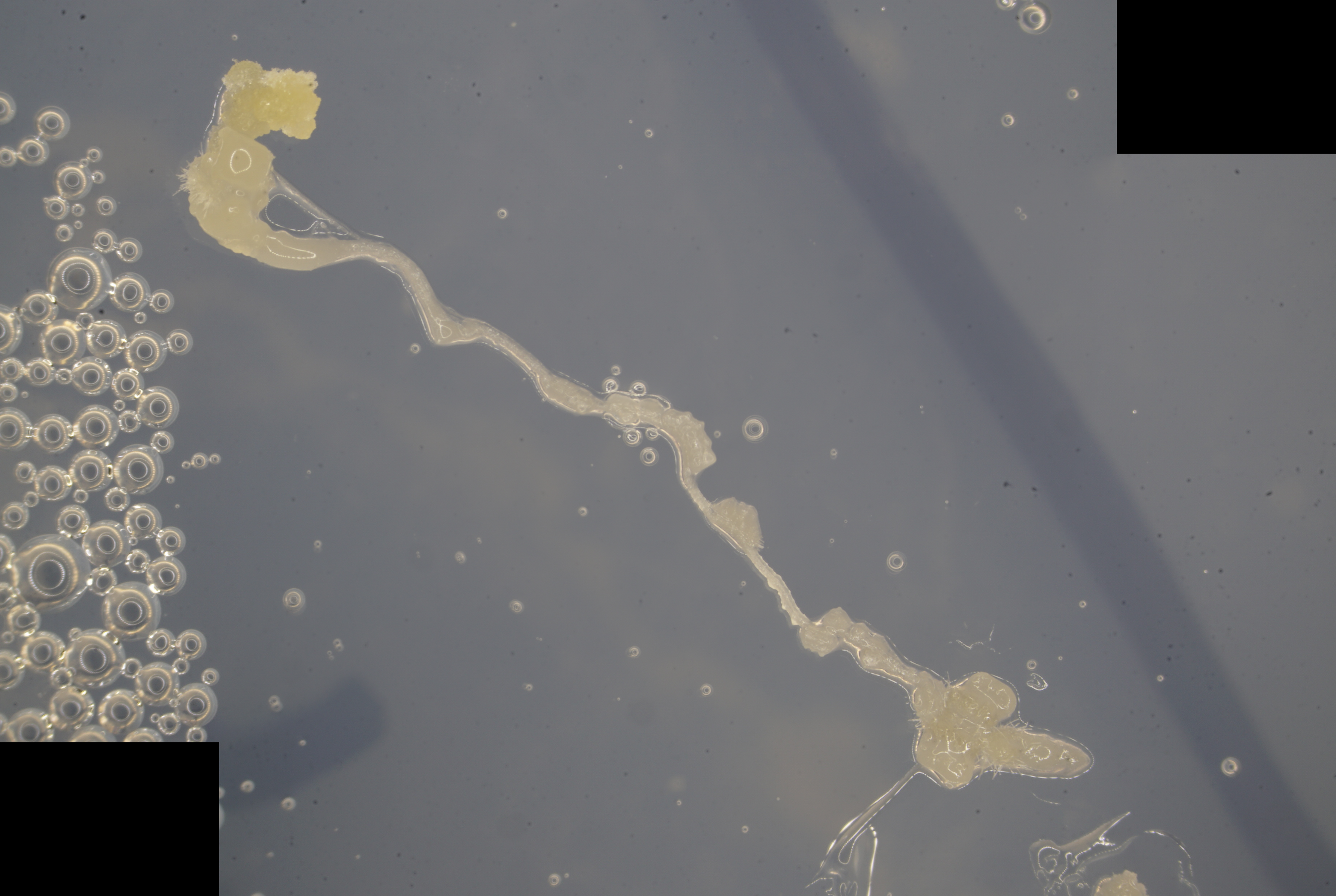

Supplement: Supplementary file 4 — Source data Fig. 3 [file 44319_2025_433_MOESM4_ESM.zip › Fig 3/3A/col brz1.tif]

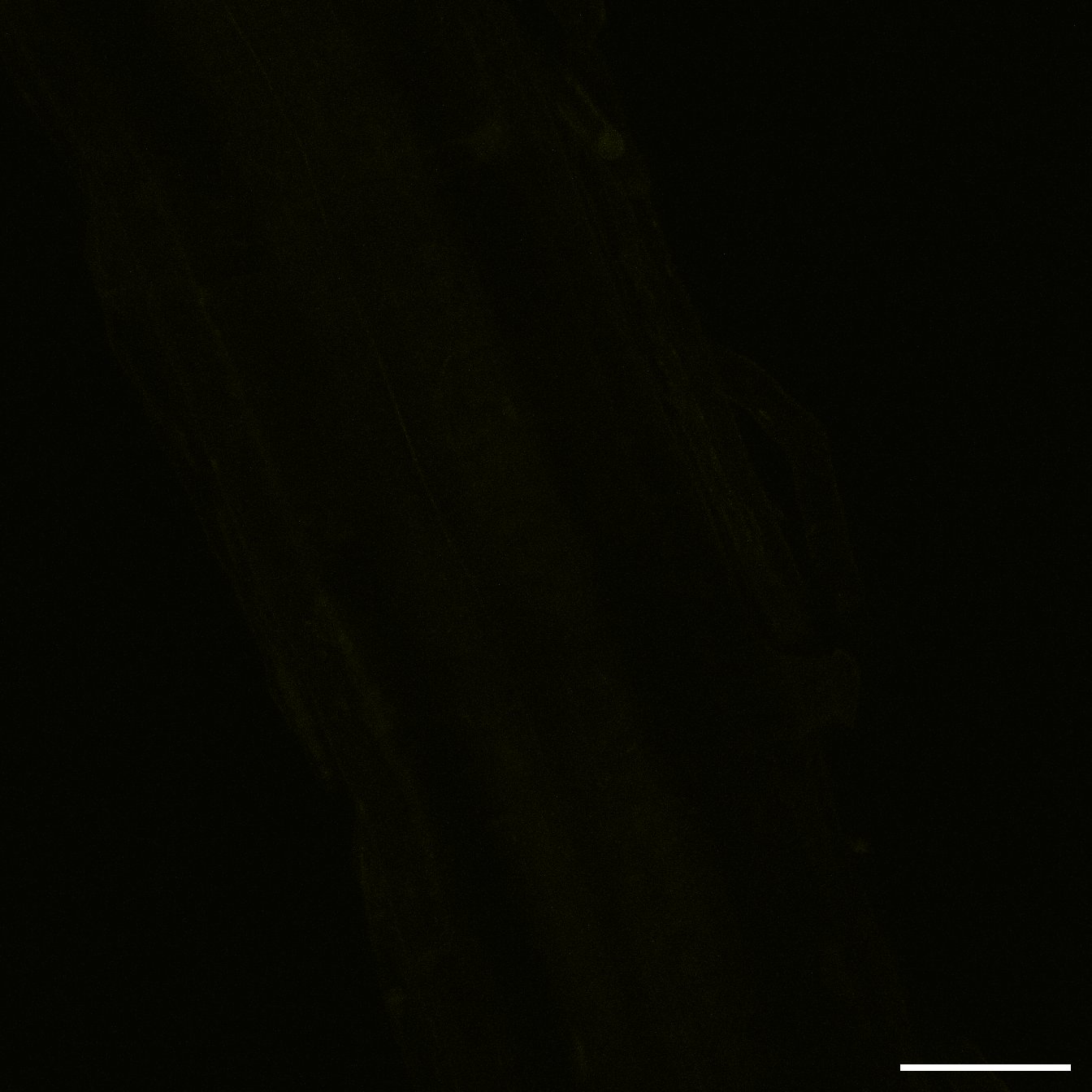

Supplement: Supplementary file 5 — Source data Fig. 4 [file 44319_2025_433_MOESM5_ESM.zip › Fig 4/4A/CIM/BES1-YFP CIM mz/C1-S11 BES CIM Image 41.jpg]

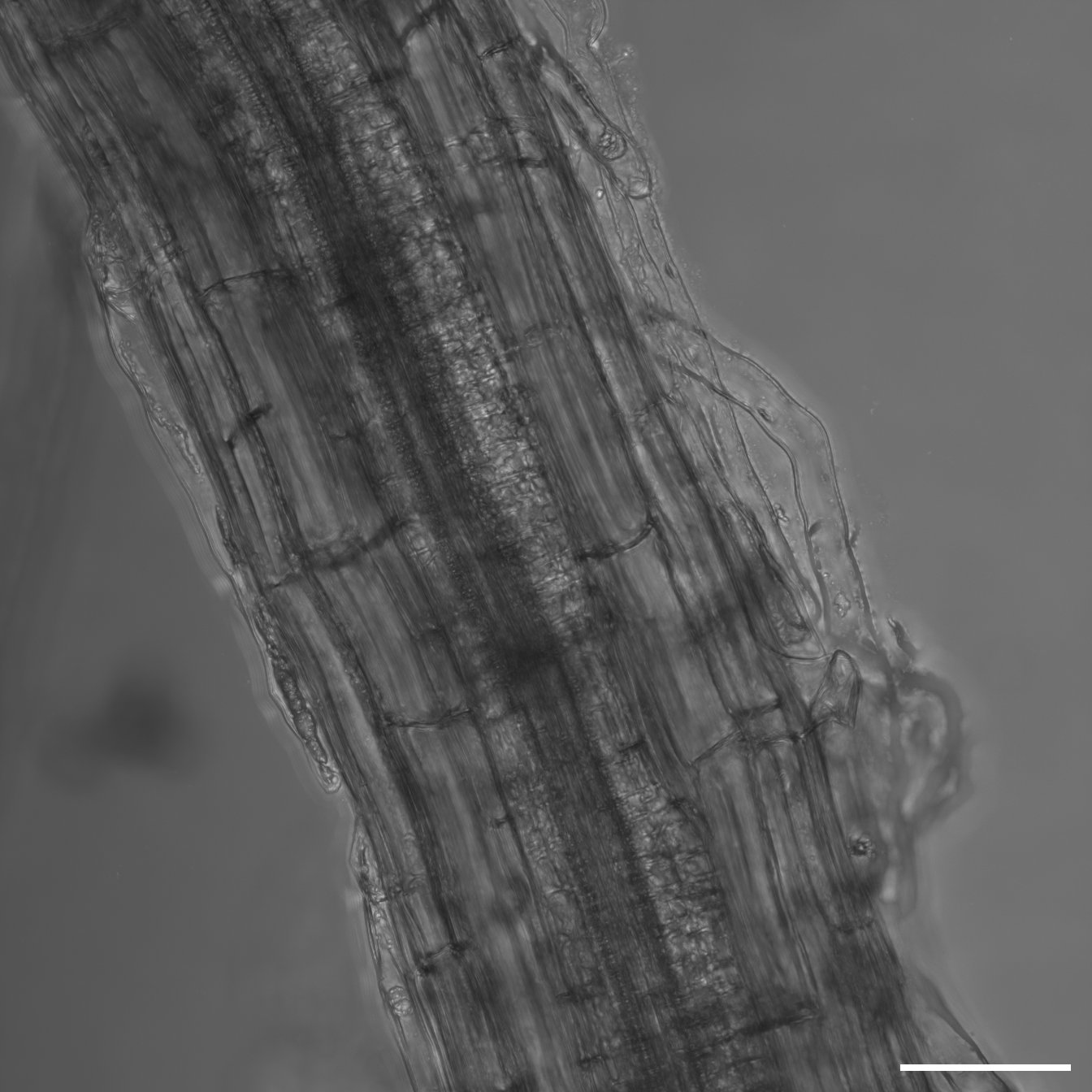

Supplement: Supplementary file 5 — Source data Fig. 4 [file 44319_2025_433_MOESM5_ESM.zip › Fig 4/4A/CIM/BES1-YFP CIM mz/C2-S11 BES CIM Image 41.jpg]

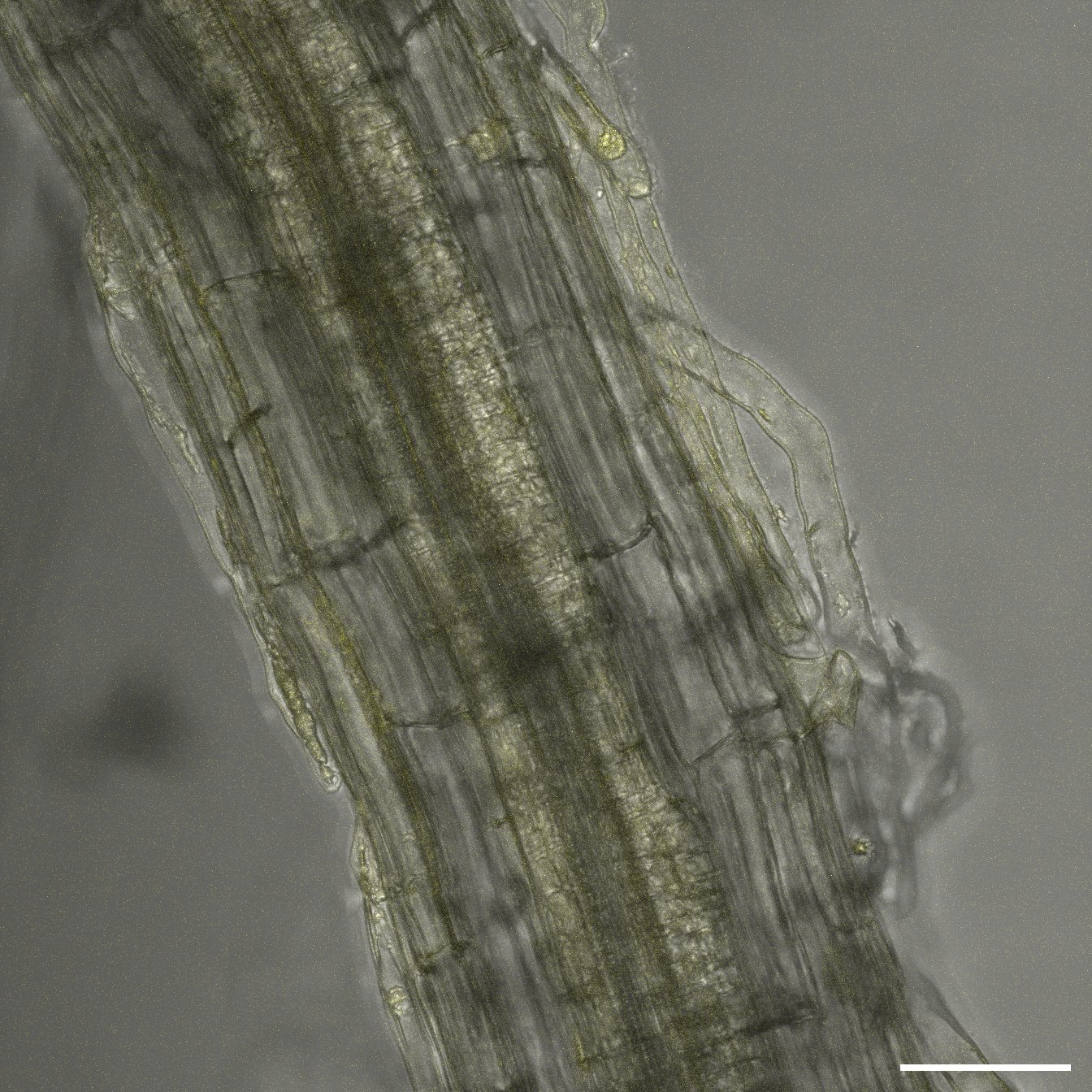

Supplement: Supplementary file 5 — Source data Fig. 4 [file 44319_2025_433_MOESM5_ESM.zip › Fig 4/4A/CIM/BES1-YFP CIM mz/S11 BES CIM Image 41.jpg]

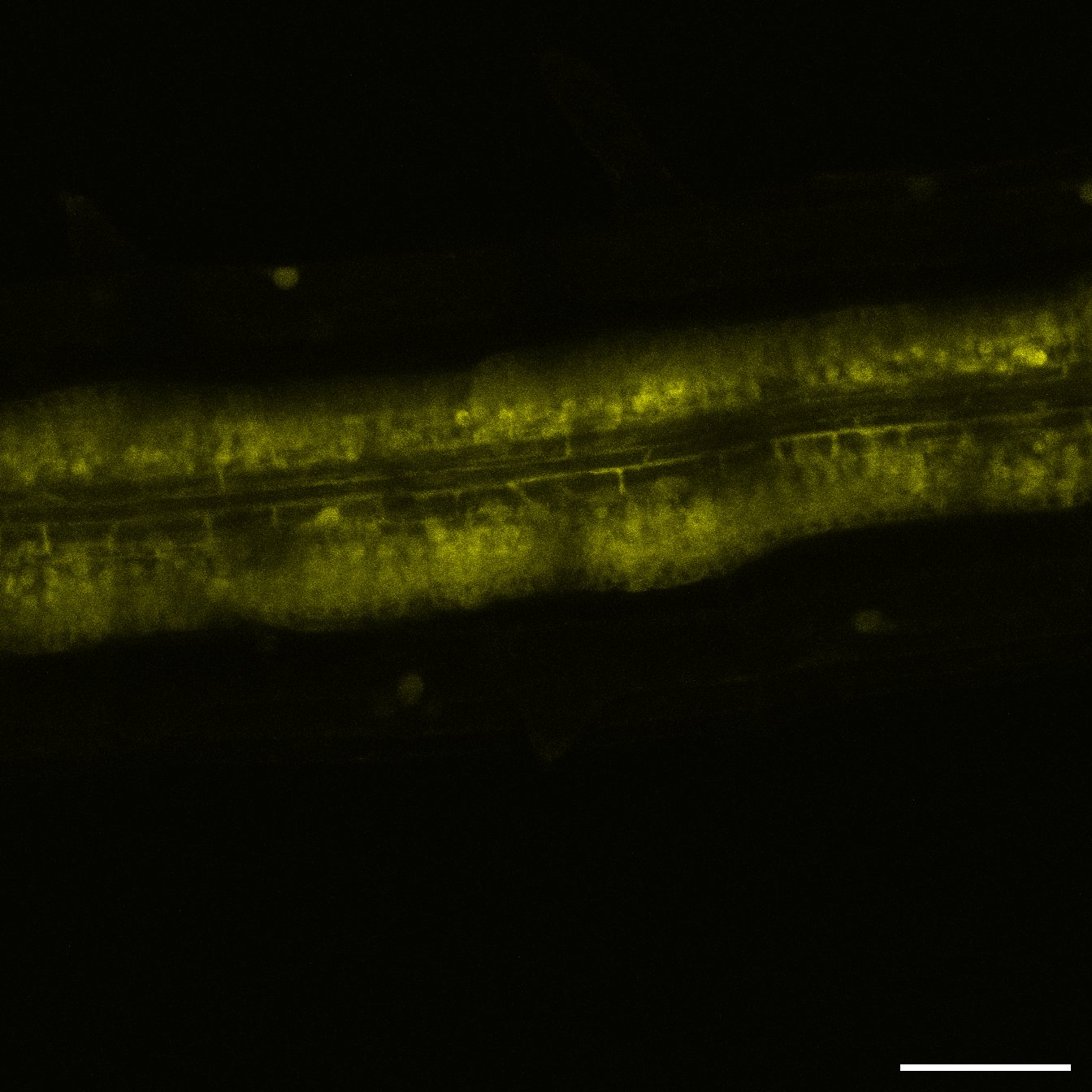

Supplement: Supplementary file 5 — Source data Fig. 4 [file 44319_2025_433_MOESM5_ESM.zip › Fig 4/4A/CIM/BZR1-YFP CIM mz/C1-S9 BZR1 CIM Image 13.jpg]

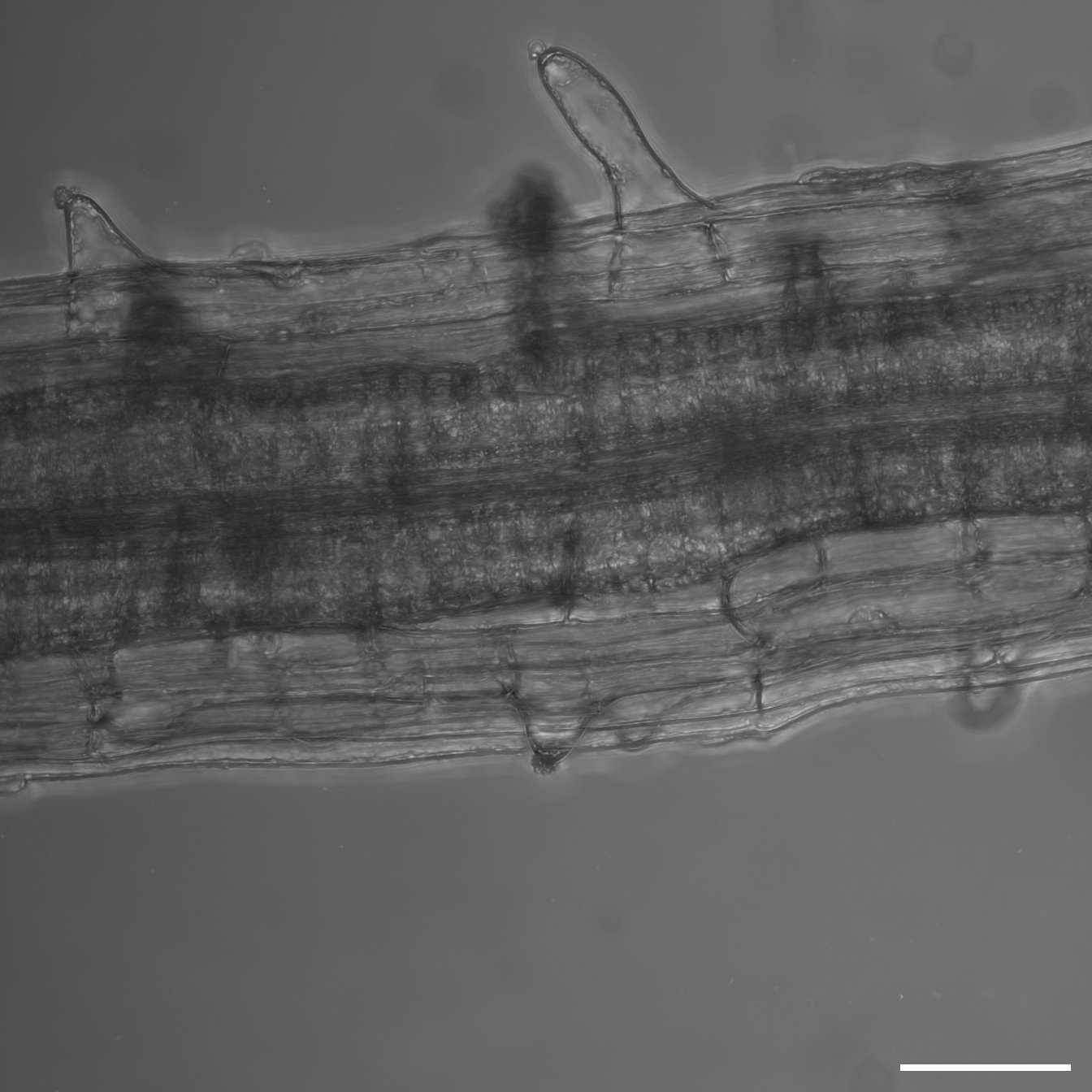

Supplement: Supplementary file 5 — Source data Fig. 4 [file 44319_2025_433_MOESM5_ESM.zip › Fig 4/4A/CIM/BZR1-YFP CIM mz/C2-S9 BZR1 CIM Image 13.jpg]

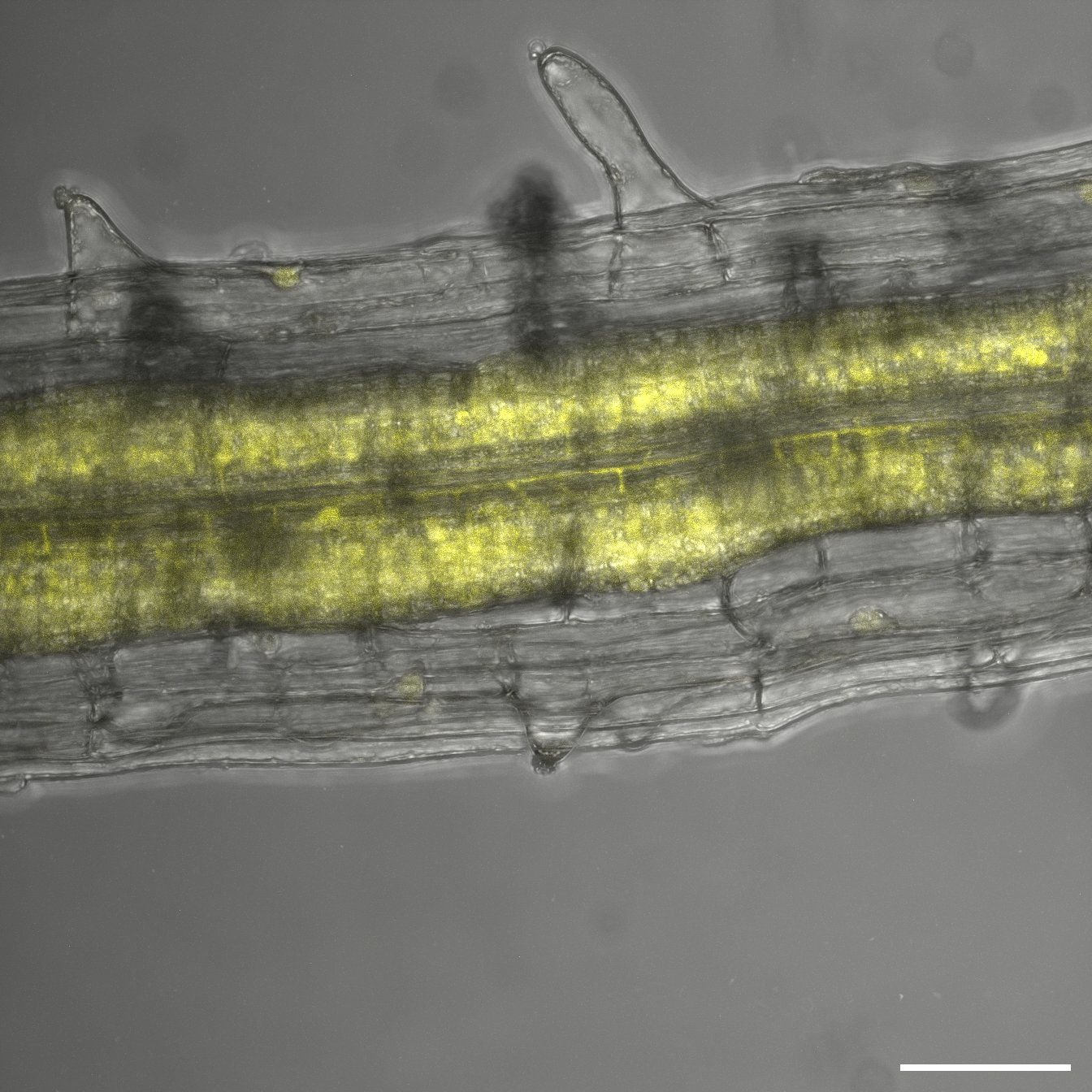

Supplement: Supplementary file 5 — Source data Fig. 4 [file 44319_2025_433_MOESM5_ESM.zip › Fig 4/4A/CIM/BZR1-YFP CIM mz/S9 BZR1 CIM Image 13.jpg]

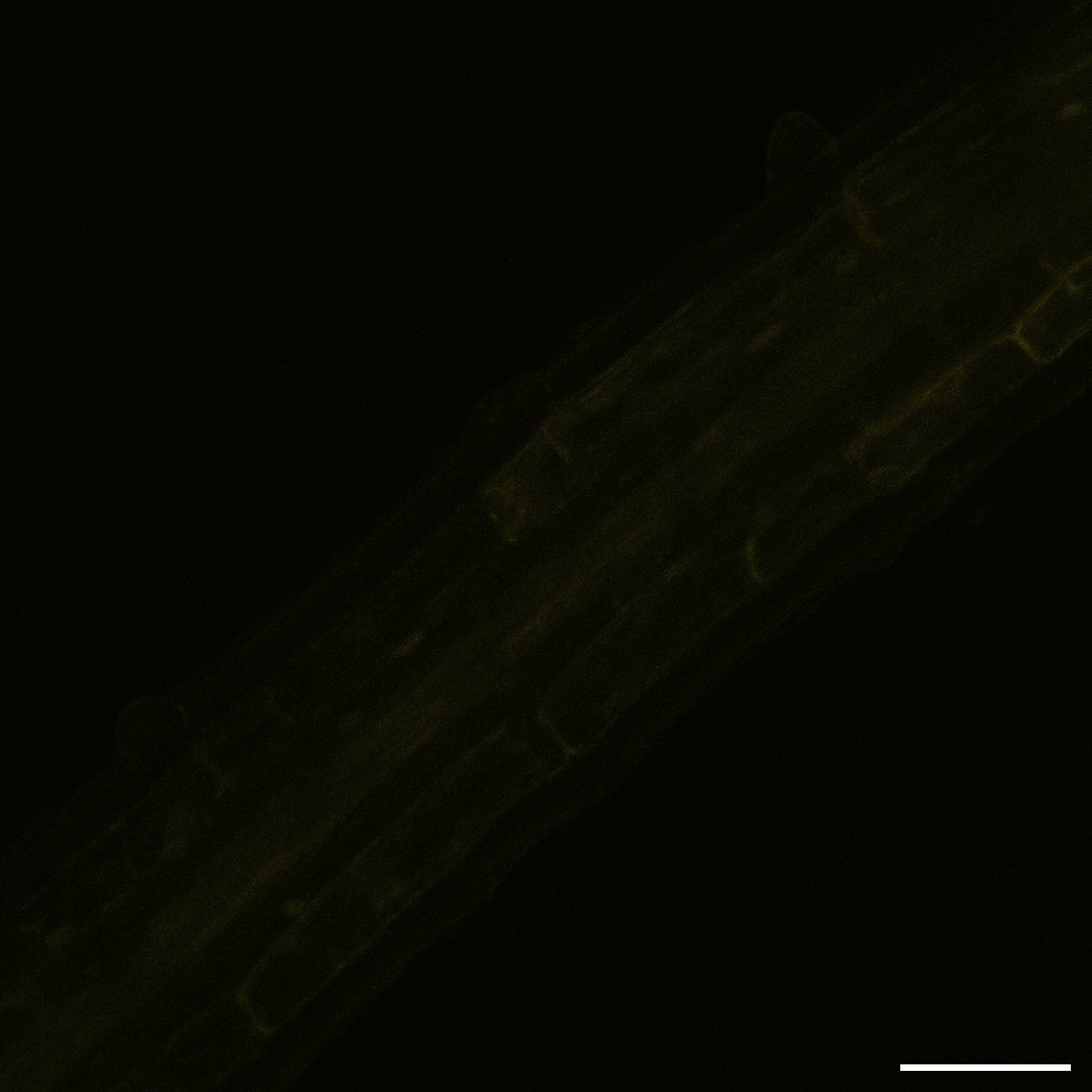

Supplement: Supplementary file 5 — Source data Fig. 4 [file 44319_2025_433_MOESM5_ESM.zip › Fig 4/4A/NT/BES1-YFP NT mz/C1-S4 BES1 Control Image 46.jpg]

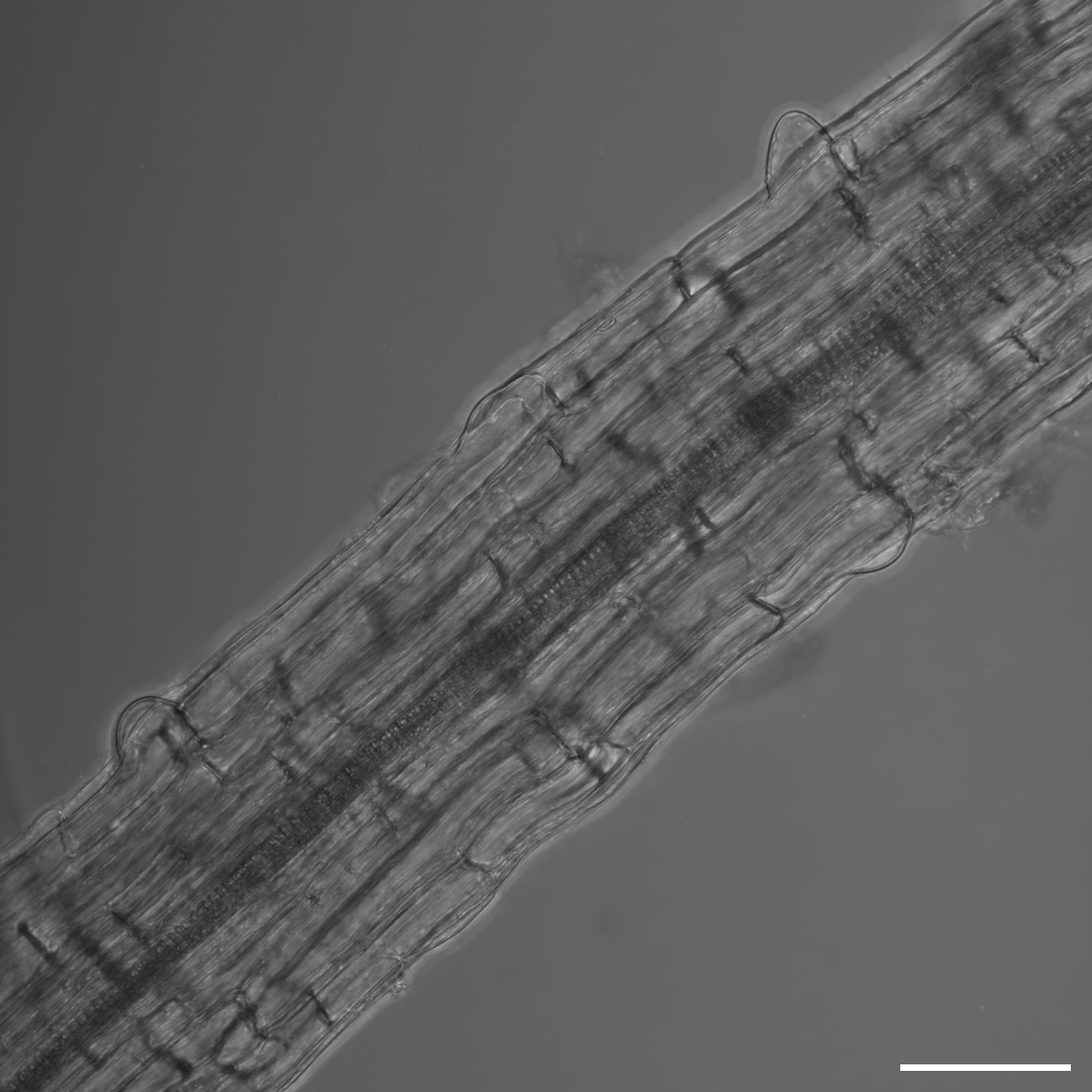

Supplement: Supplementary file 5 — Source data Fig. 4 [file 44319_2025_433_MOESM5_ESM.zip › Fig 4/4A/NT/BES1-YFP NT mz/C2-S4 BES1 Control Image 46.jpg]

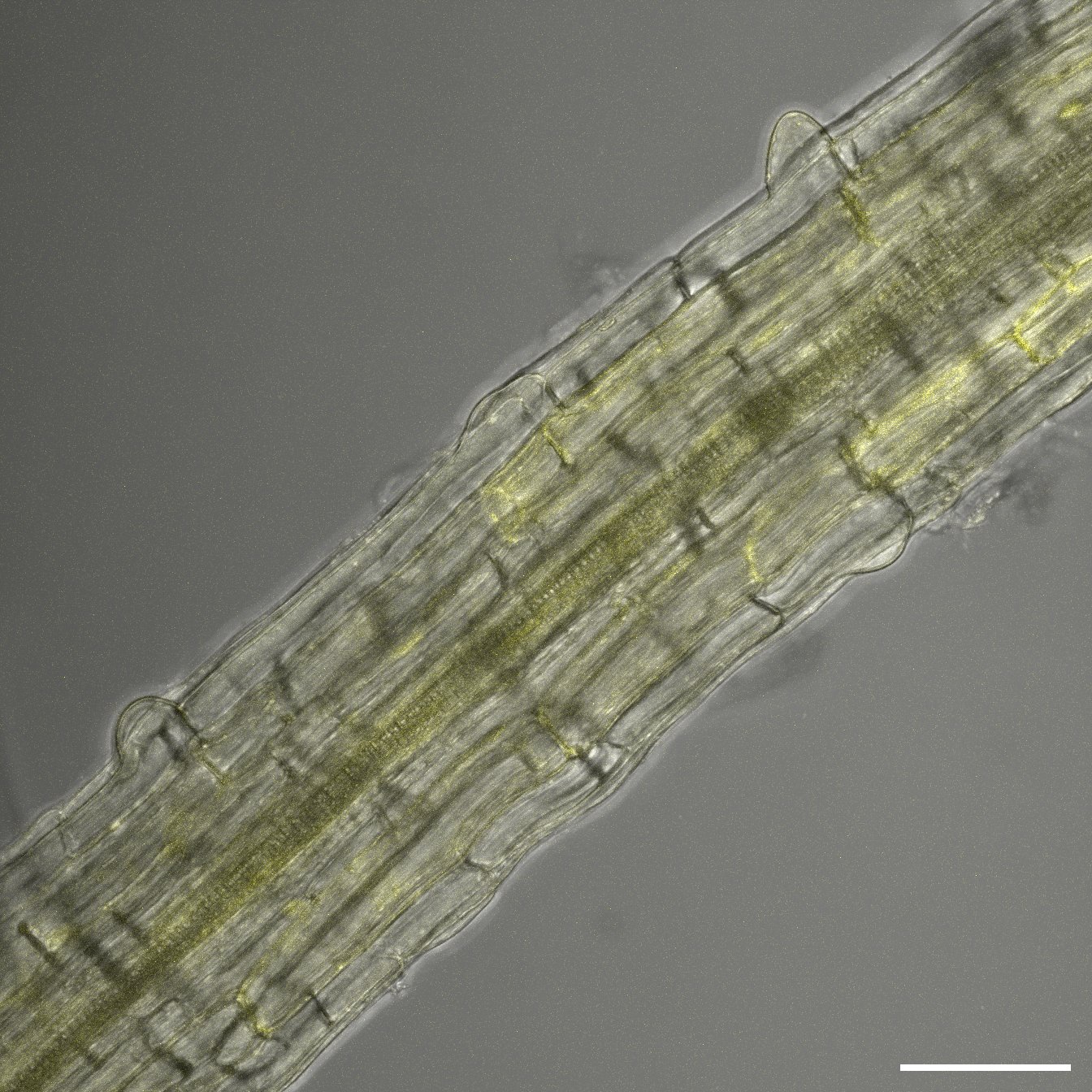

Supplement: Supplementary file 5 — Source data Fig. 4 [file 44319_2025_433_MOESM5_ESM.zip › Fig 4/4A/NT/BES1-YFP NT mz/S4 BES1 Control Image 46.jpg]

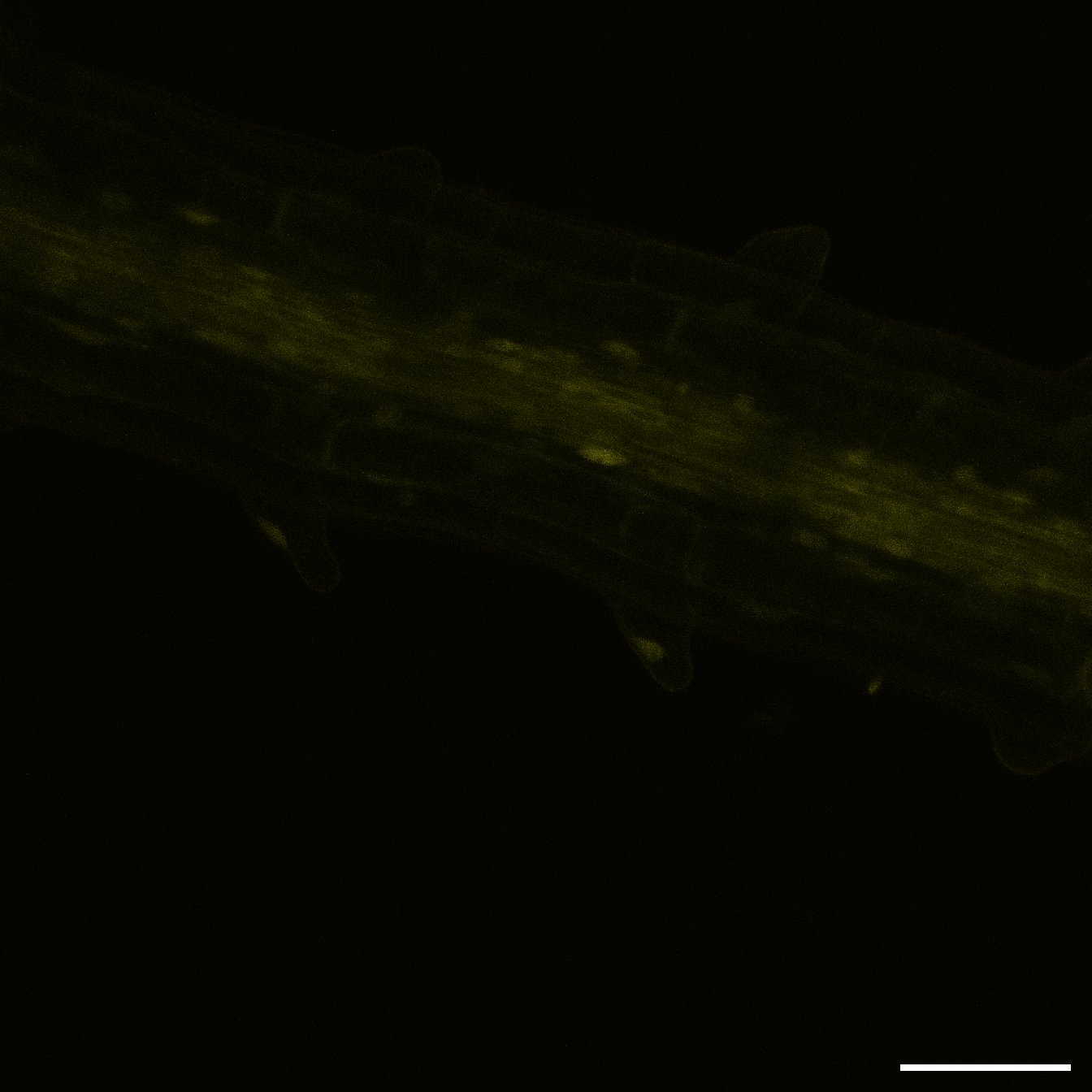

Supplement: Supplementary file 5 — Source data Fig. 4 [file 44319_2025_433_MOESM5_ESM.zip › Fig 4/4A/NT/BZR1-YFP NT mz/C1-S3 BZR1 Control Image 17.jpg]

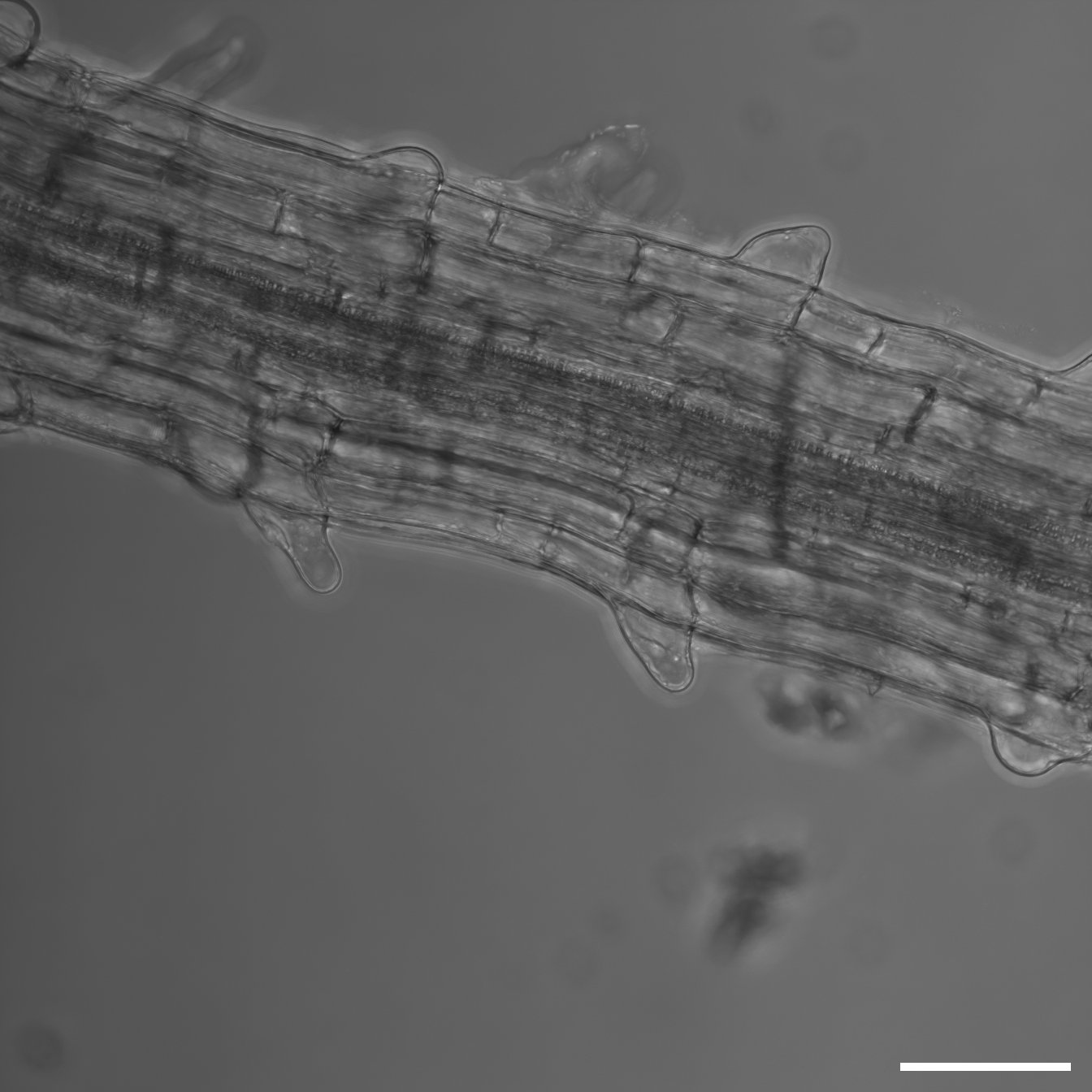

Supplement: Supplementary file 5 — Source data Fig. 4 [file 44319_2025_433_MOESM5_ESM.zip › Fig 4/4A/NT/BZR1-YFP NT mz/C2-S3 BZR1 Control Image 17.jpg]

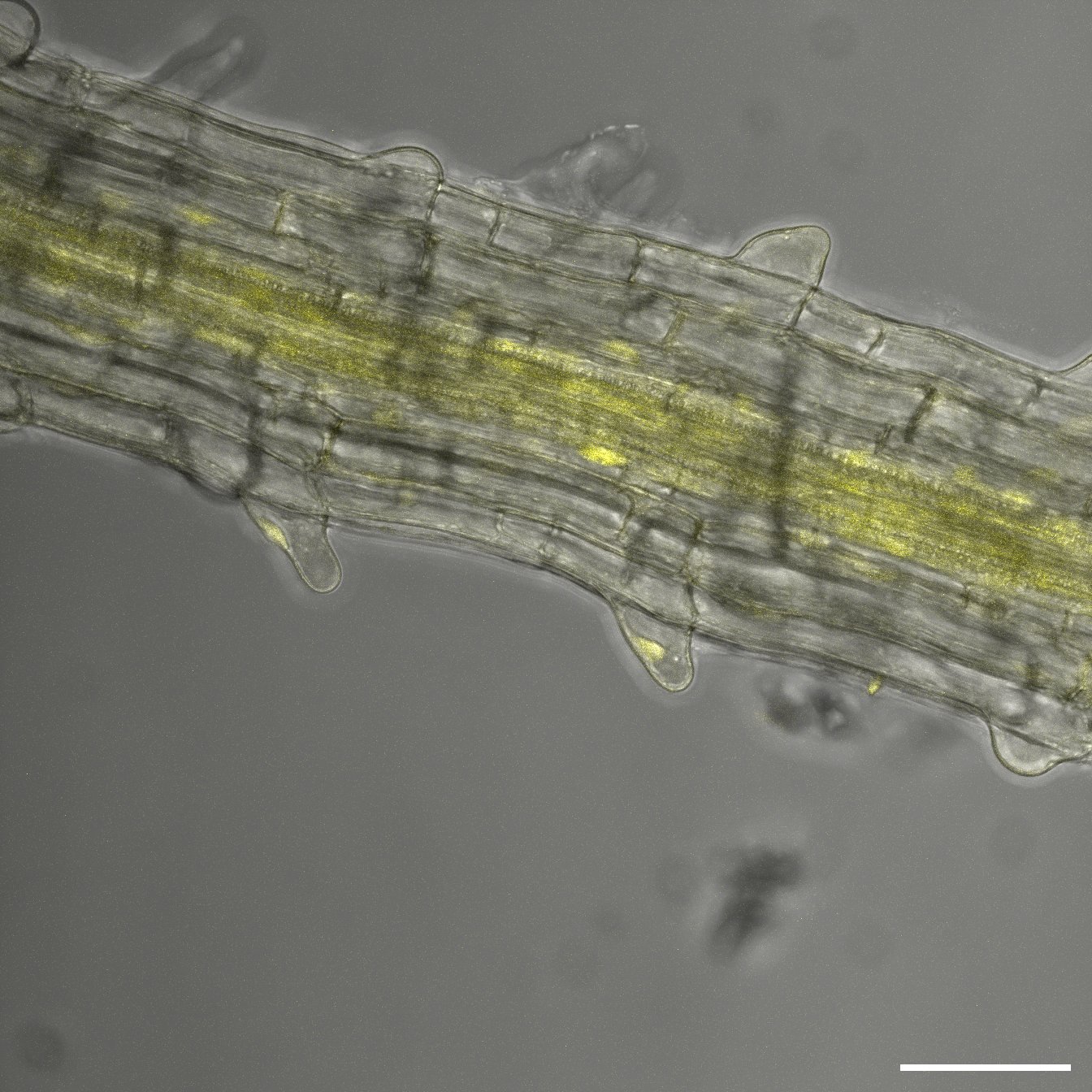

Supplement: Supplementary file 5 — Source data Fig. 4 [file 44319_2025_433_MOESM5_ESM.zip › Fig 4/4A/NT/BZR1-YFP NT mz/S3 BZR1 Control Image 17.jpg]

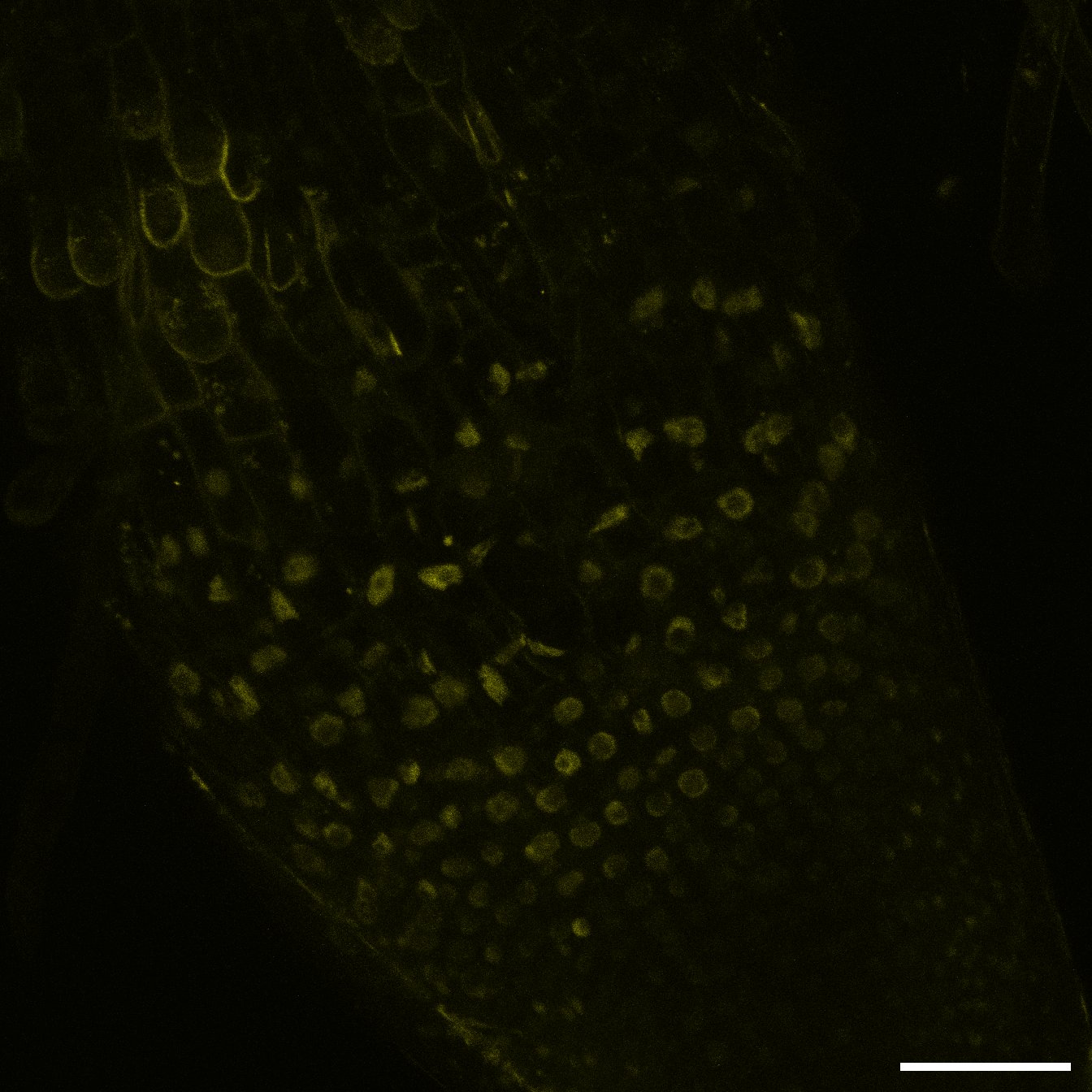

Supplement: Supplementary file 5 — Source data Fig. 4 [file 44319_2025_433_MOESM5_ESM.zip › Fig 4/4A/SIM/BES1-YFP SIM/C1-S7 BES1-YFP SIM Image 89_Maximum intensity projection.jpg]

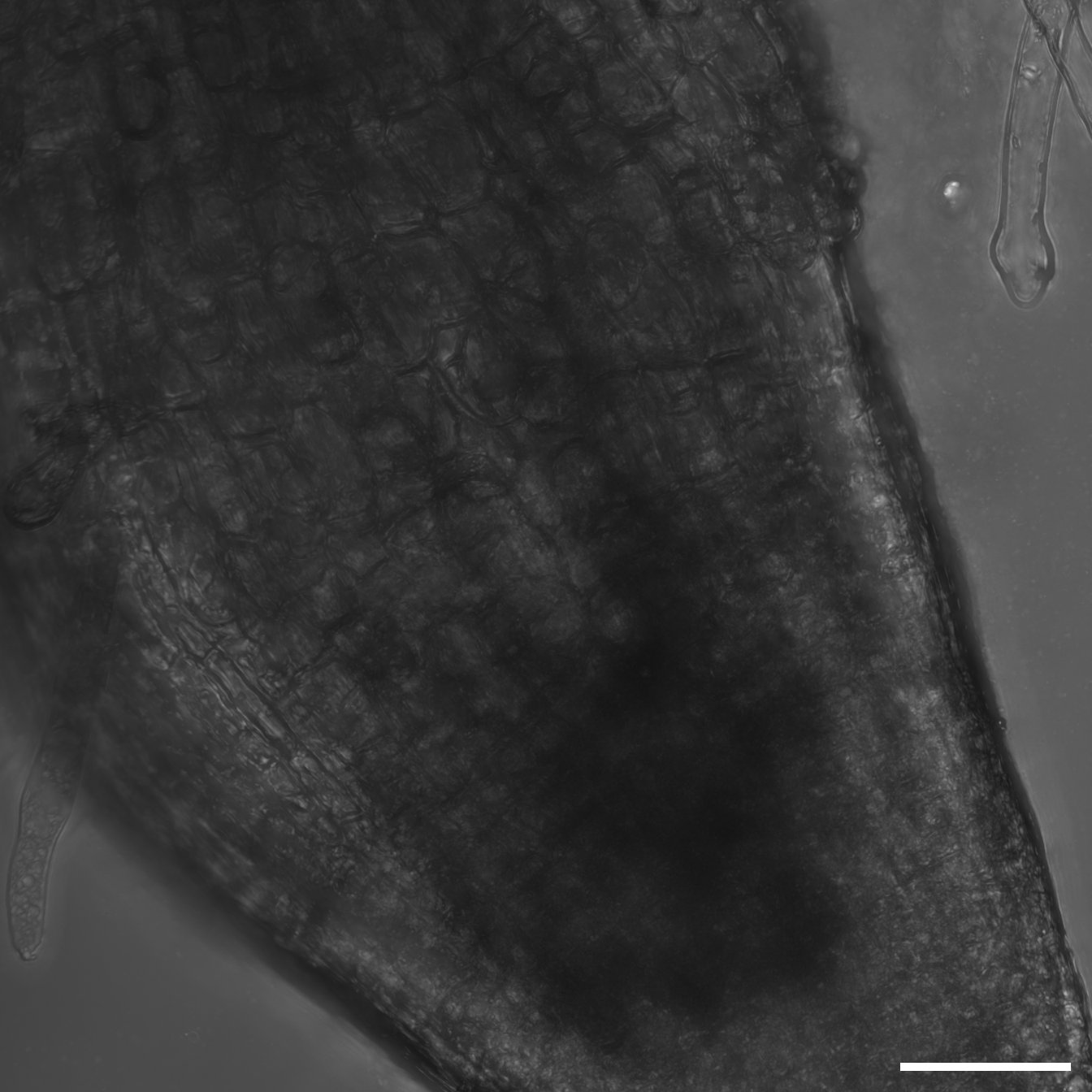

Supplement: Supplementary file 5 — Source data Fig. 4 [file 44319_2025_433_MOESM5_ESM.zip › Fig 4/4A/SIM/BES1-YFP SIM/C2-S7 BES1-YFP SIM Image 89_Maximum intensity projection.jpg]

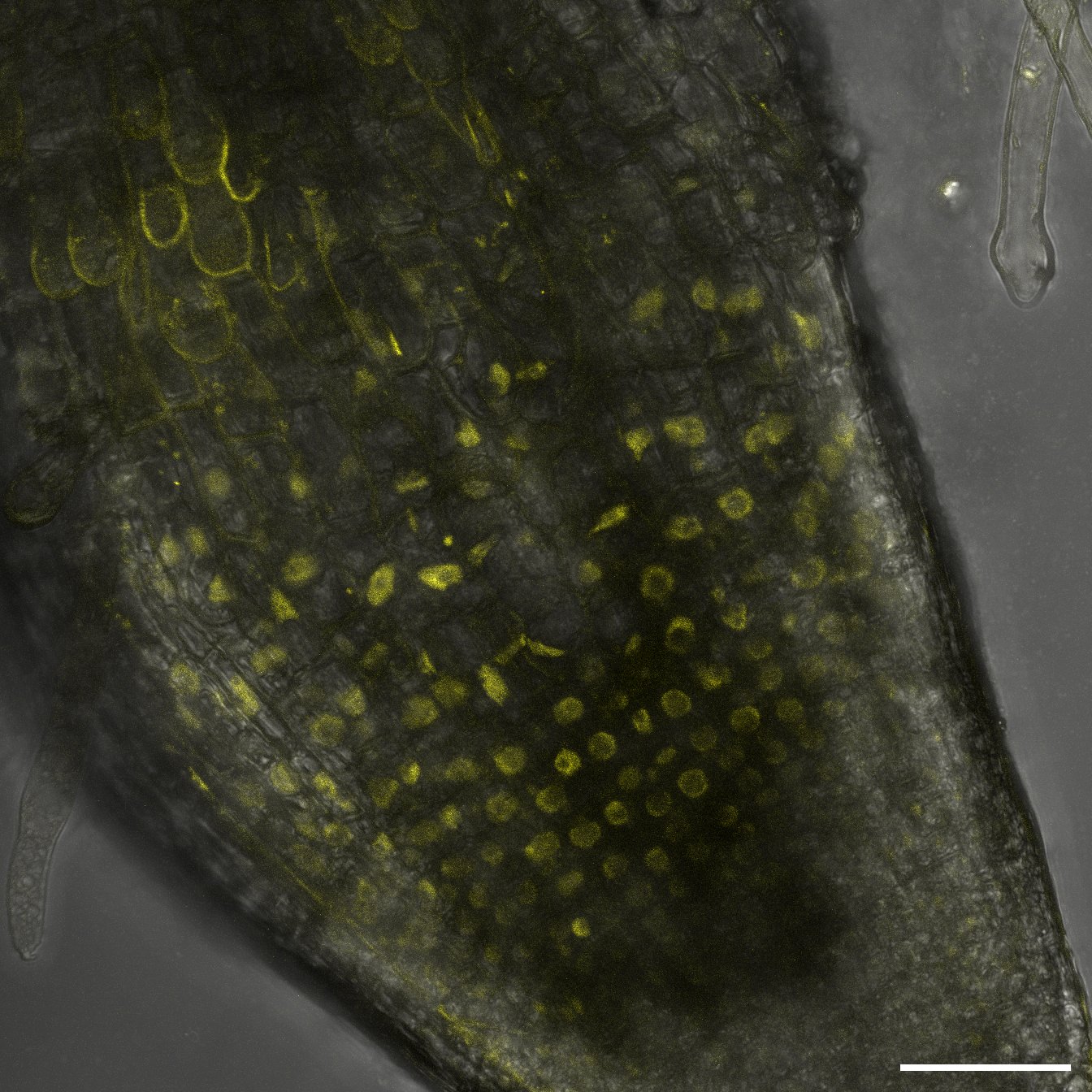

Supplement: Supplementary file 5 — Source data Fig. 4 [file 44319_2025_433_MOESM5_ESM.zip › Fig 4/4A/SIM/BES1-YFP SIM/S7 BES1-YFP SIM Image 89_Maximum intensity projection.jpg]

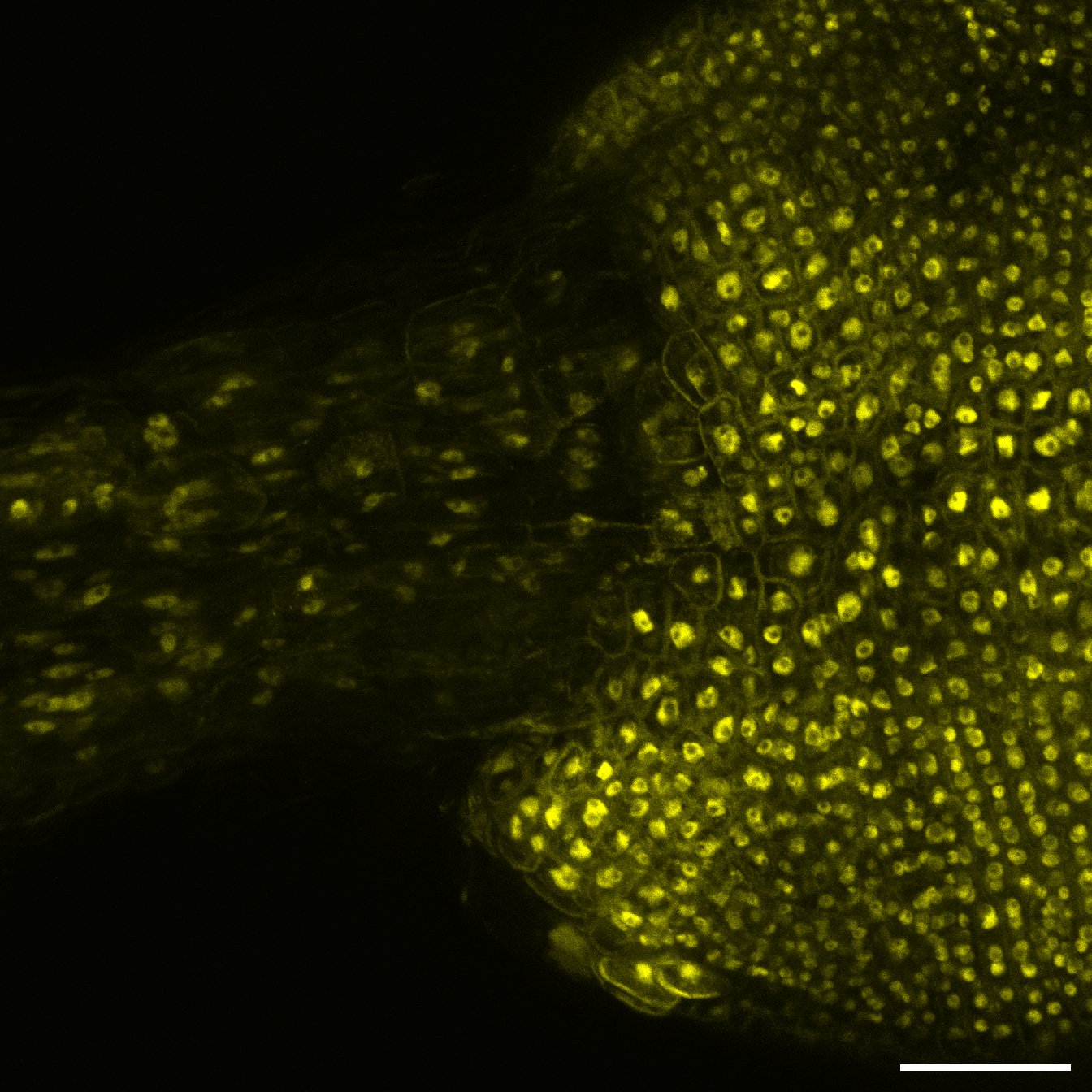

Supplement: Supplementary file 5 — Source data Fig. 4 [file 44319_2025_433_MOESM5_ESM.zip › Fig 4/4A/SIM/BZR1-YFP SIM/C1-S4 BZR1-YFP SIM Image 63_Maximum intensity projection.jpg]

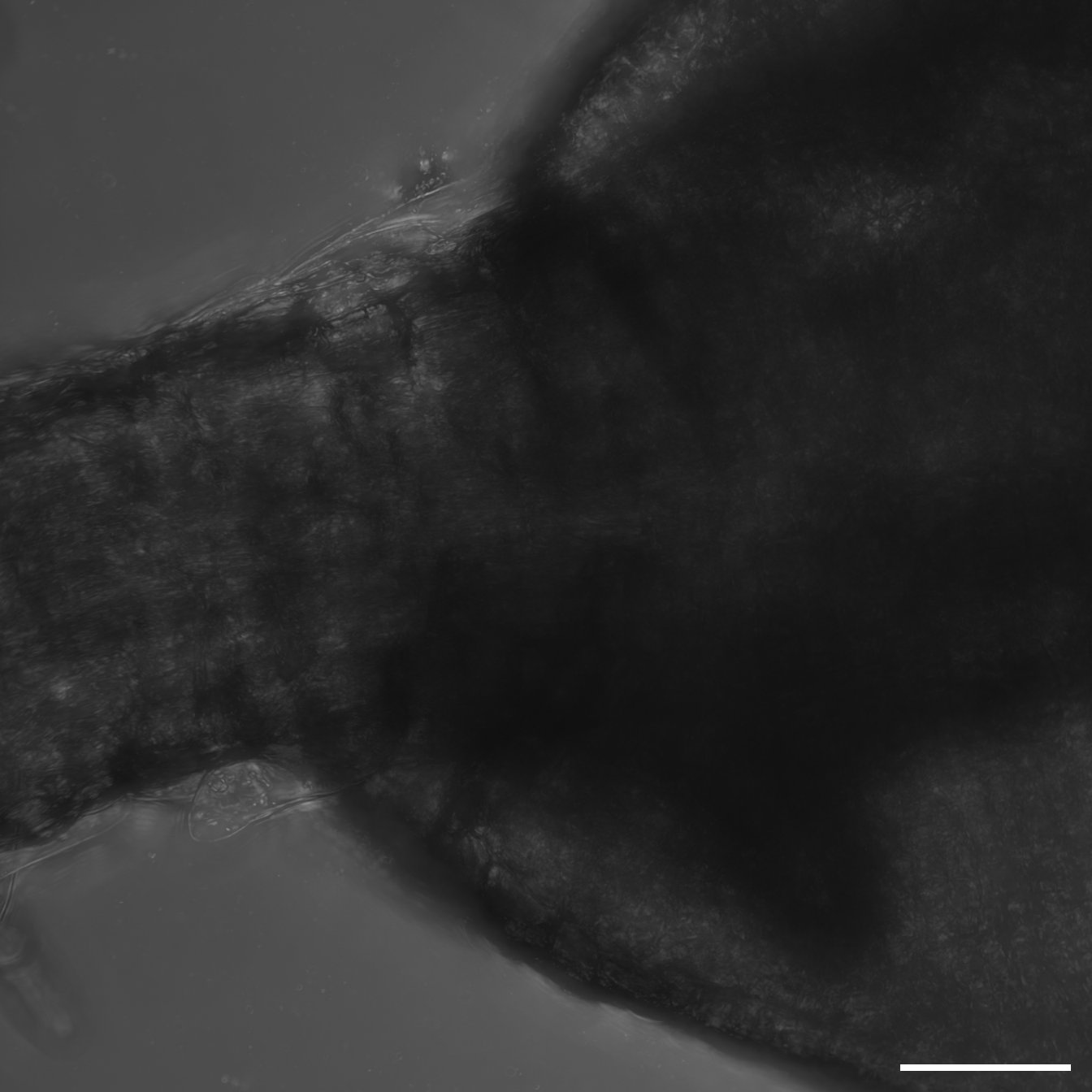

Supplement: Supplementary file 5 — Source data Fig. 4 [file 44319_2025_433_MOESM5_ESM.zip › Fig 4/4A/SIM/BZR1-YFP SIM/C2-S4 BZR1-YFP SIM Image 63_Maximum intensity projection.jpg]

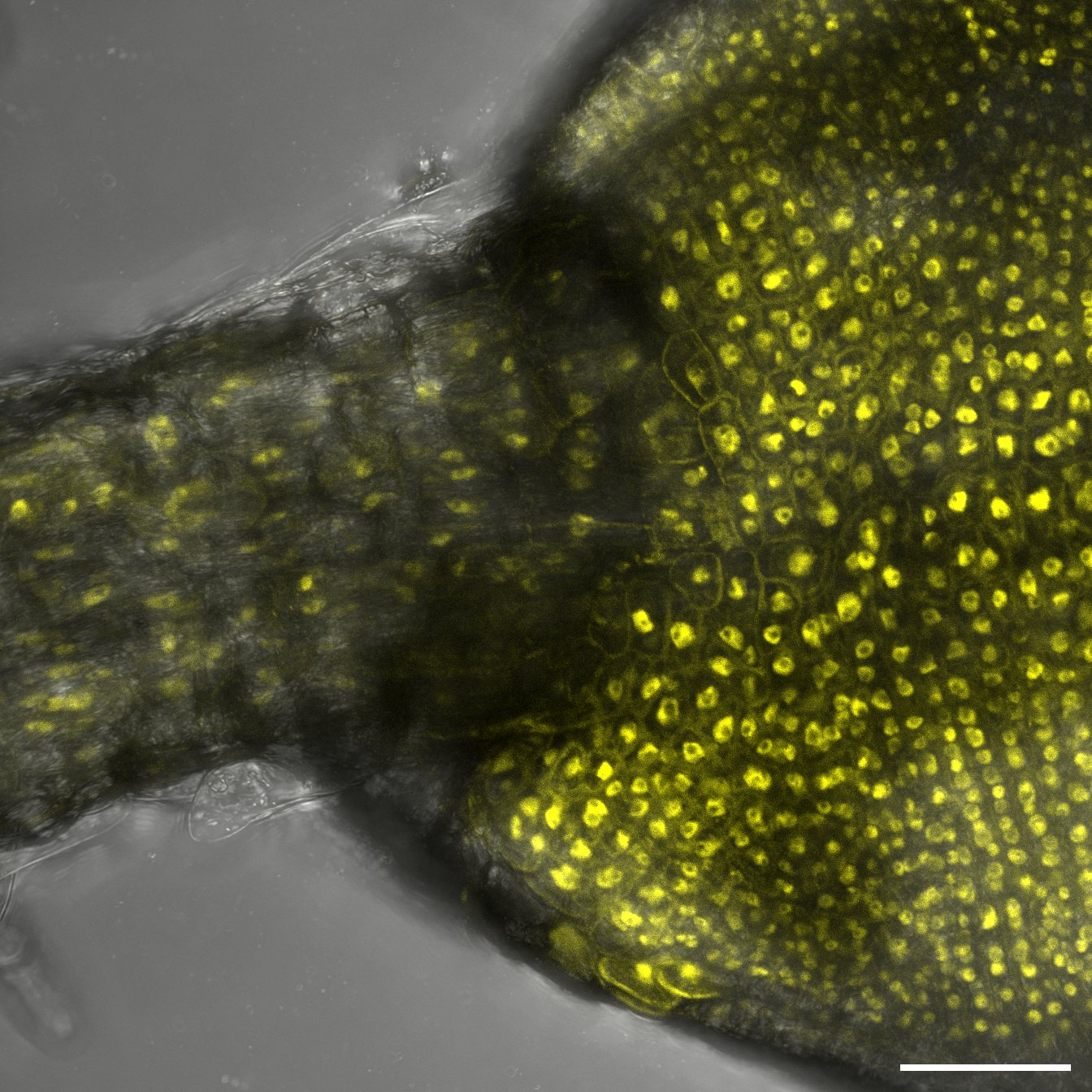

Supplement: Supplementary file 5 — Source data Fig. 4 [file 44319_2025_433_MOESM5_ESM.zip › Fig 4/4A/SIM/BZR1-YFP SIM/S4 BZR1-YFP SIM Image 63_Maximum intensity projection.jpg]

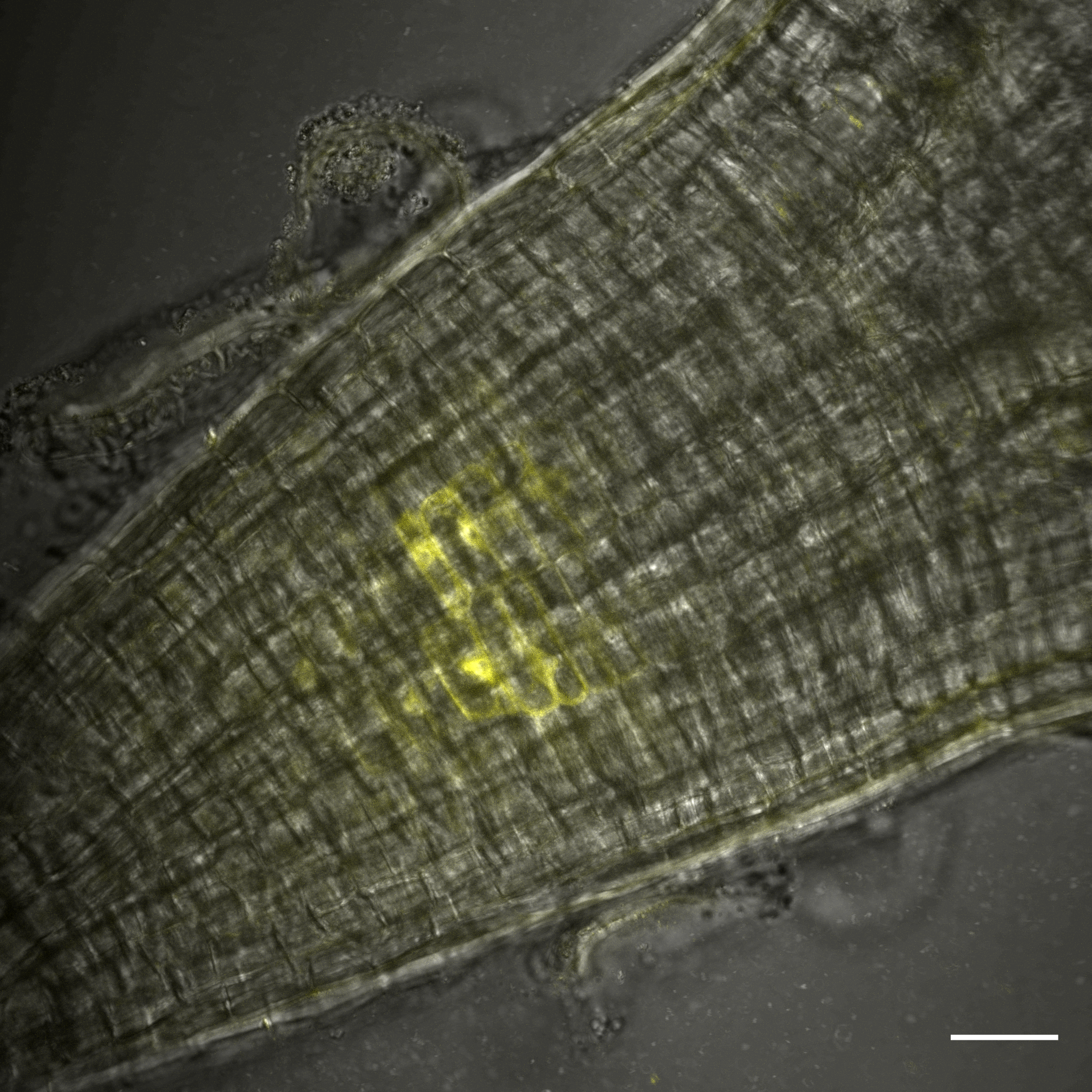

Supplement: Supplementary file 5 — Source data Fig. 4 [file 44319_2025_433_MOESM5_ESM.zip › Fig 4/4D/CIM/BES1-YFP CIM rt/Image 37_Maximum intensity projection_c1-2.tif]

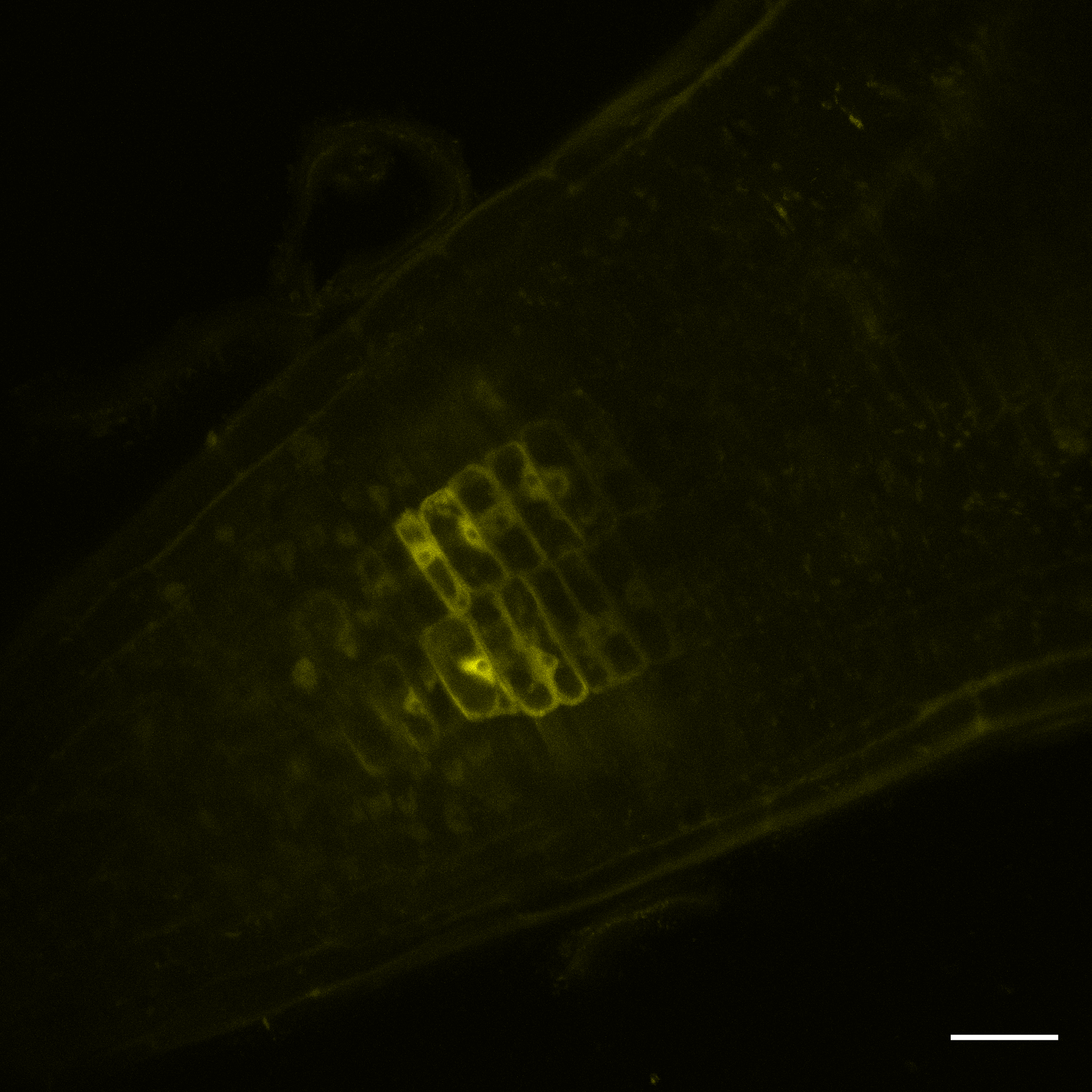

Supplement: Supplementary file 5 — Source data Fig. 4 [file 44319_2025_433_MOESM5_ESM.zip › Fig 4/4D/CIM/BES1-YFP CIM rt/Image 37_Maximum intensity projection_c1.tif]

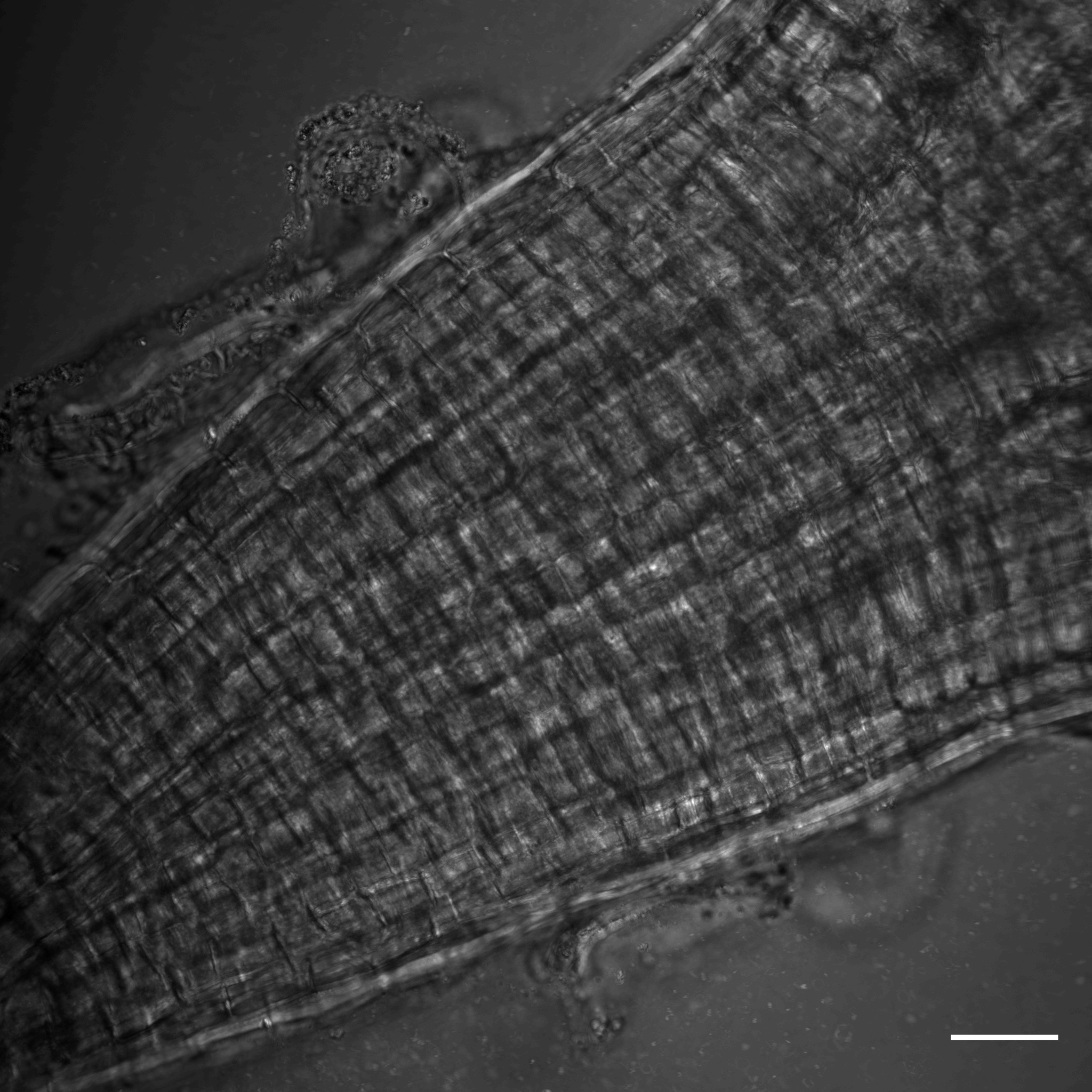

Supplement: Supplementary file 5 — Source data Fig. 4 [file 44319_2025_433_MOESM5_ESM.zip › Fig 4/4D/CIM/BES1-YFP CIM rt/Image 37_Maximum intensity projection_c2.tif]

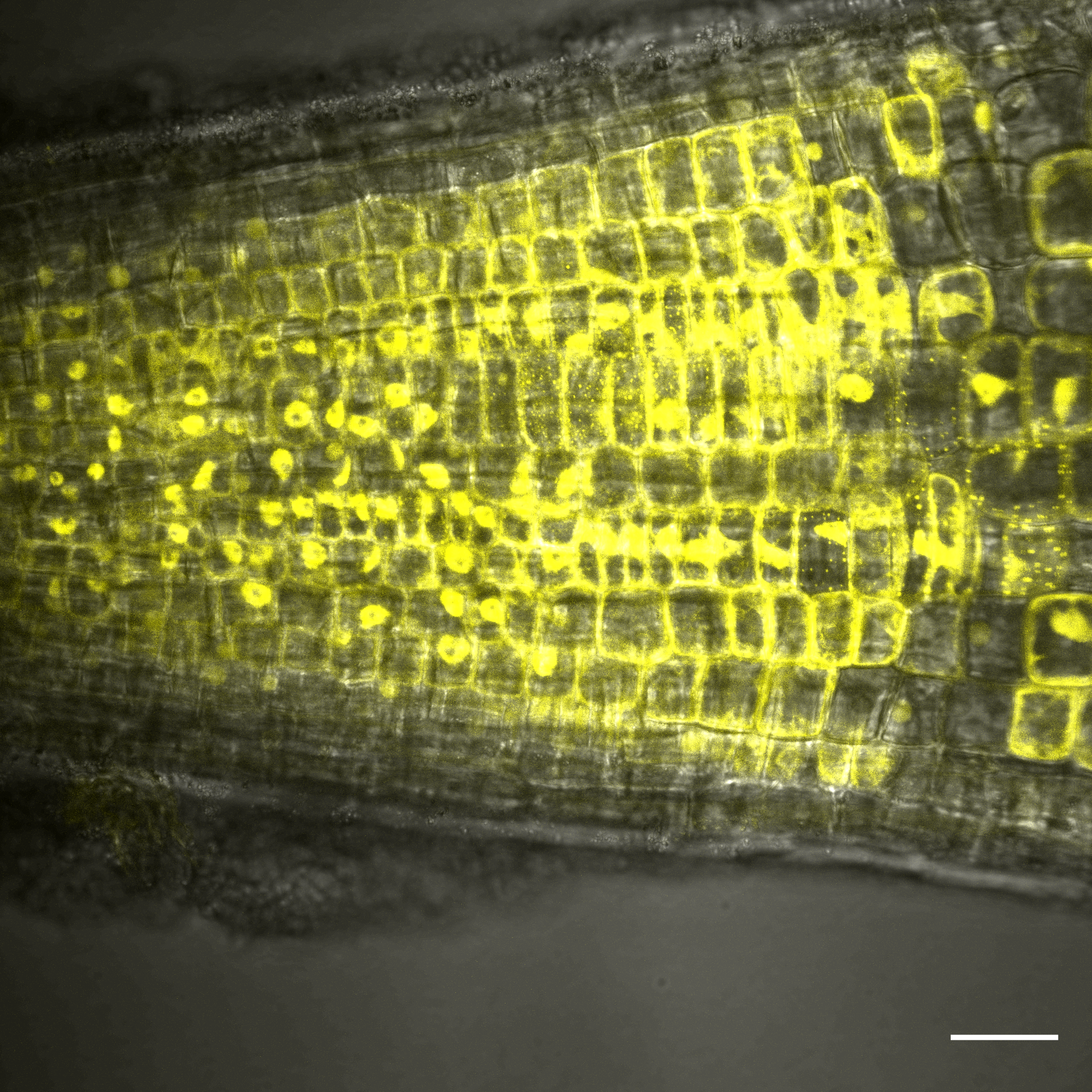

Supplement: Supplementary file 5 — Source data Fig. 4 [file 44319_2025_433_MOESM5_ESM.zip › Fig 4/4D/CIM/BZR1-YFP CIM rt/Image 11_Maximum intensity projection_c1-2.tif]

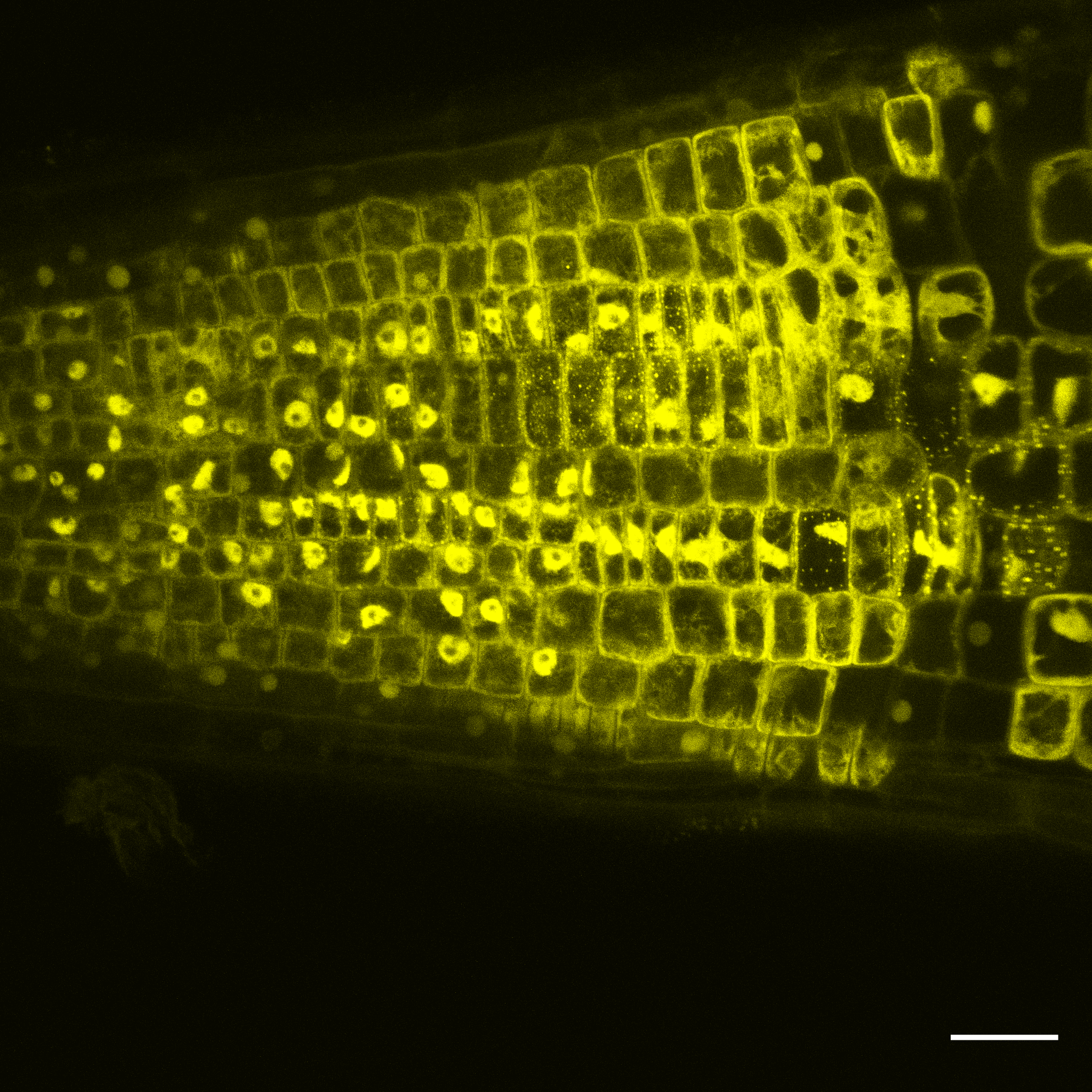

Supplement: Supplementary file 5 — Source data Fig. 4 [file 44319_2025_433_MOESM5_ESM.zip › Fig 4/4D/CIM/BZR1-YFP CIM rt/Image 11_Maximum intensity projection_c1.tif]

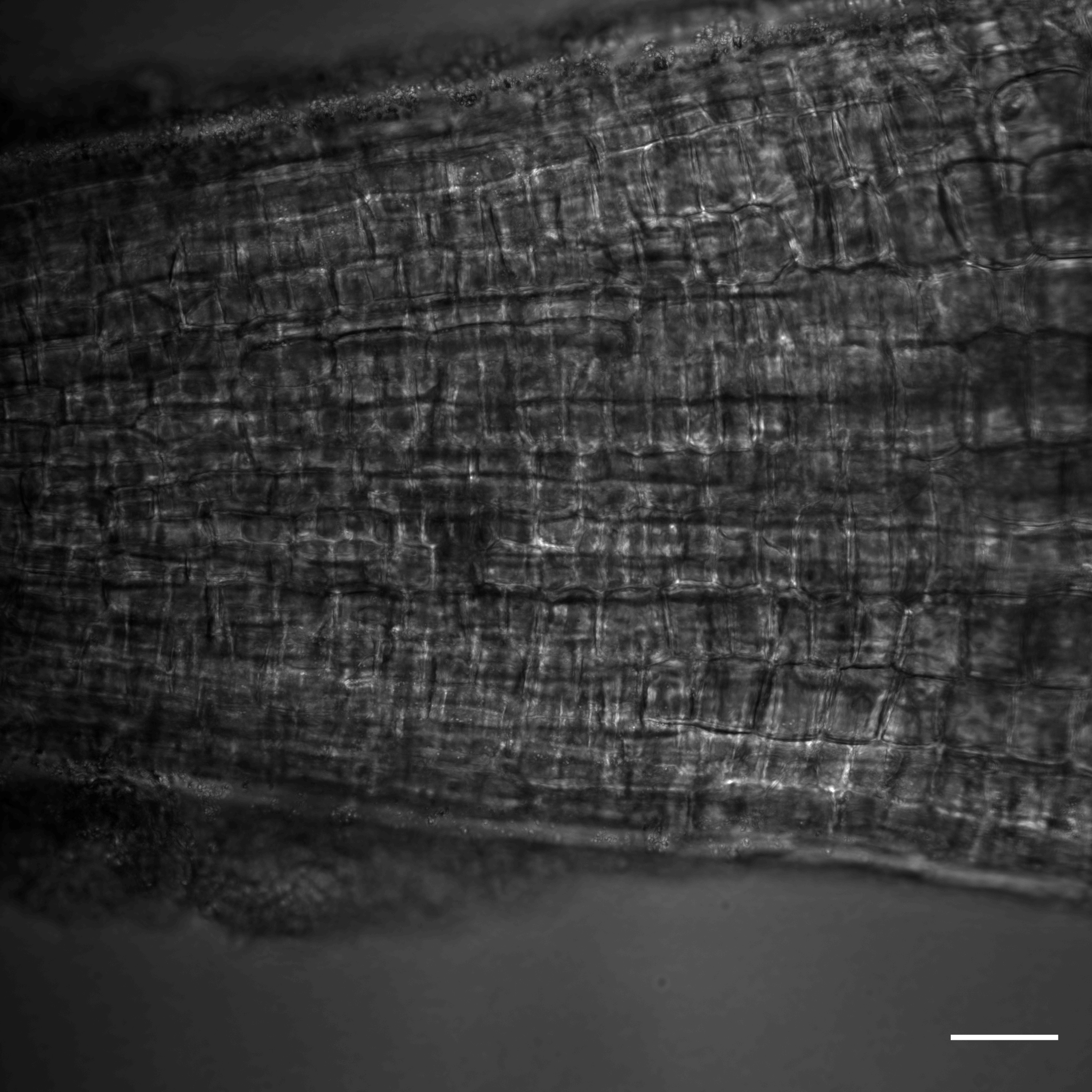

Supplement: Supplementary file 5 — Source data Fig. 4 [file 44319_2025_433_MOESM5_ESM.zip › Fig 4/4D/CIM/BZR1-YFP CIM rt/Image 11_Maximum intensity projection_c2.tif]

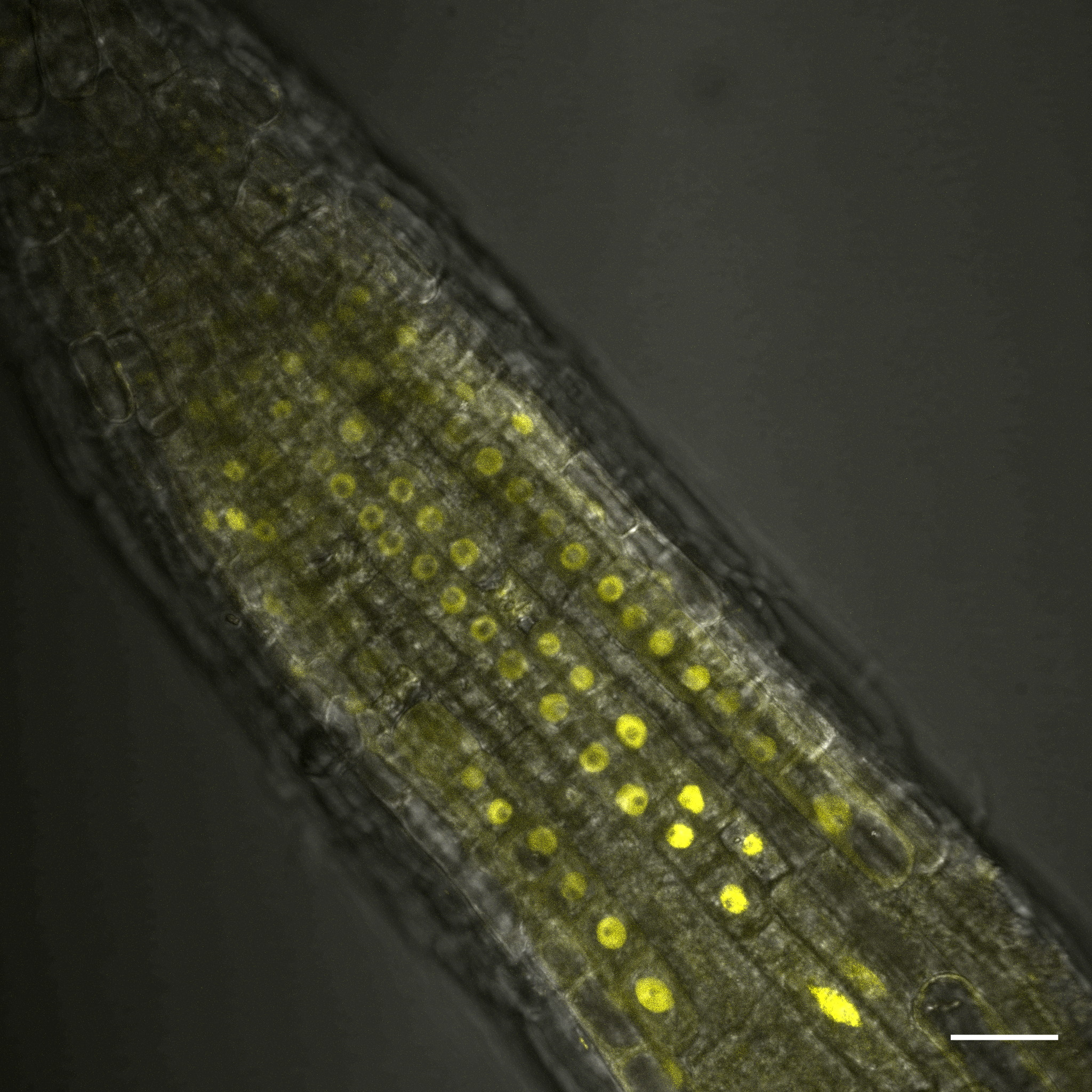

Supplement: Supplementary file 5 — Source data Fig. 4 [file 44319_2025_433_MOESM5_ESM.zip › Fig 4/4D/NT/BES1-YFP NT rt/Image 17_Maximum intensity projection_c1-2.tif]

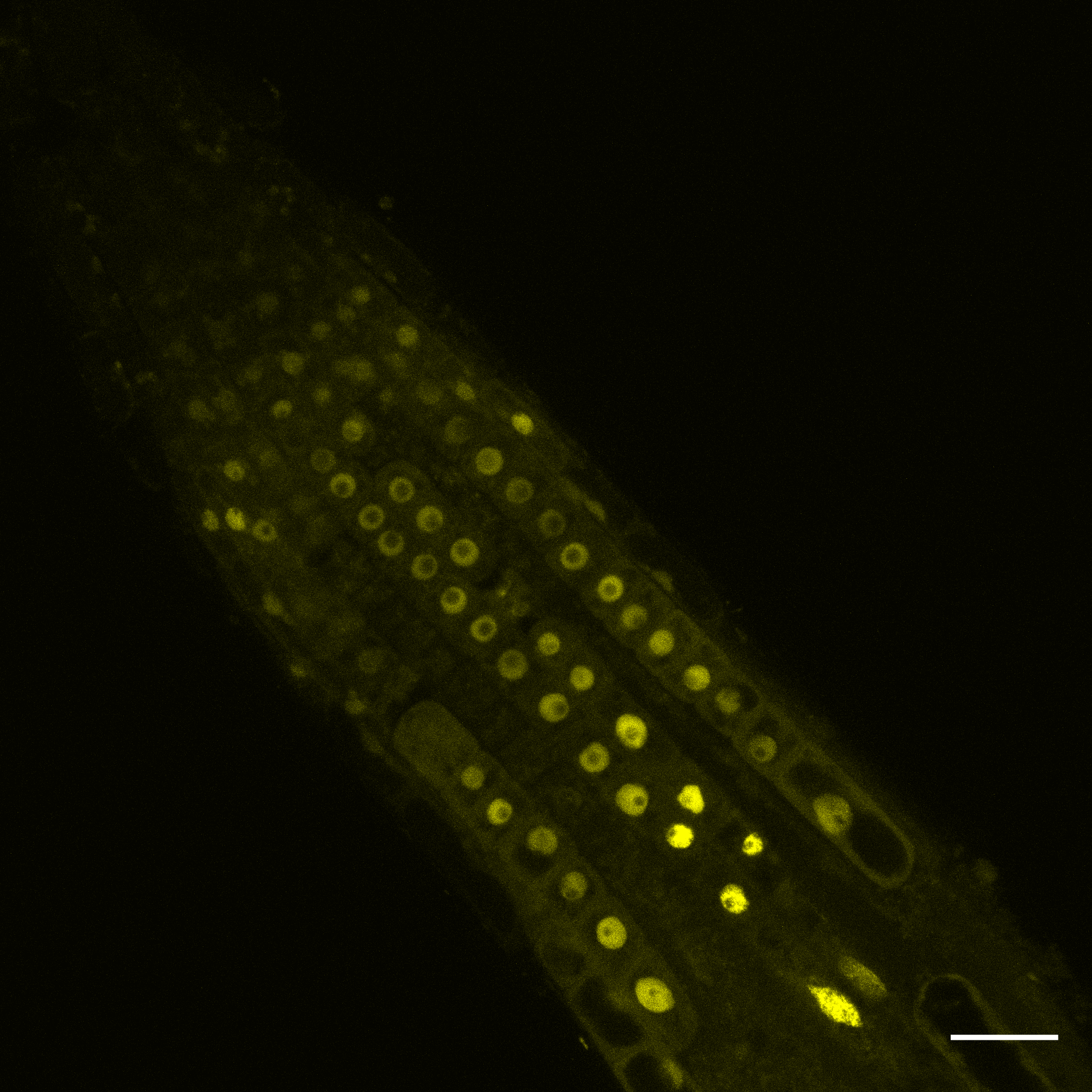

Supplement: Supplementary file 5 — Source data Fig. 4 [file 44319_2025_433_MOESM5_ESM.zip › Fig 4/4D/NT/BES1-YFP NT rt/Image 17_Maximum intensity projection_c1.tif]

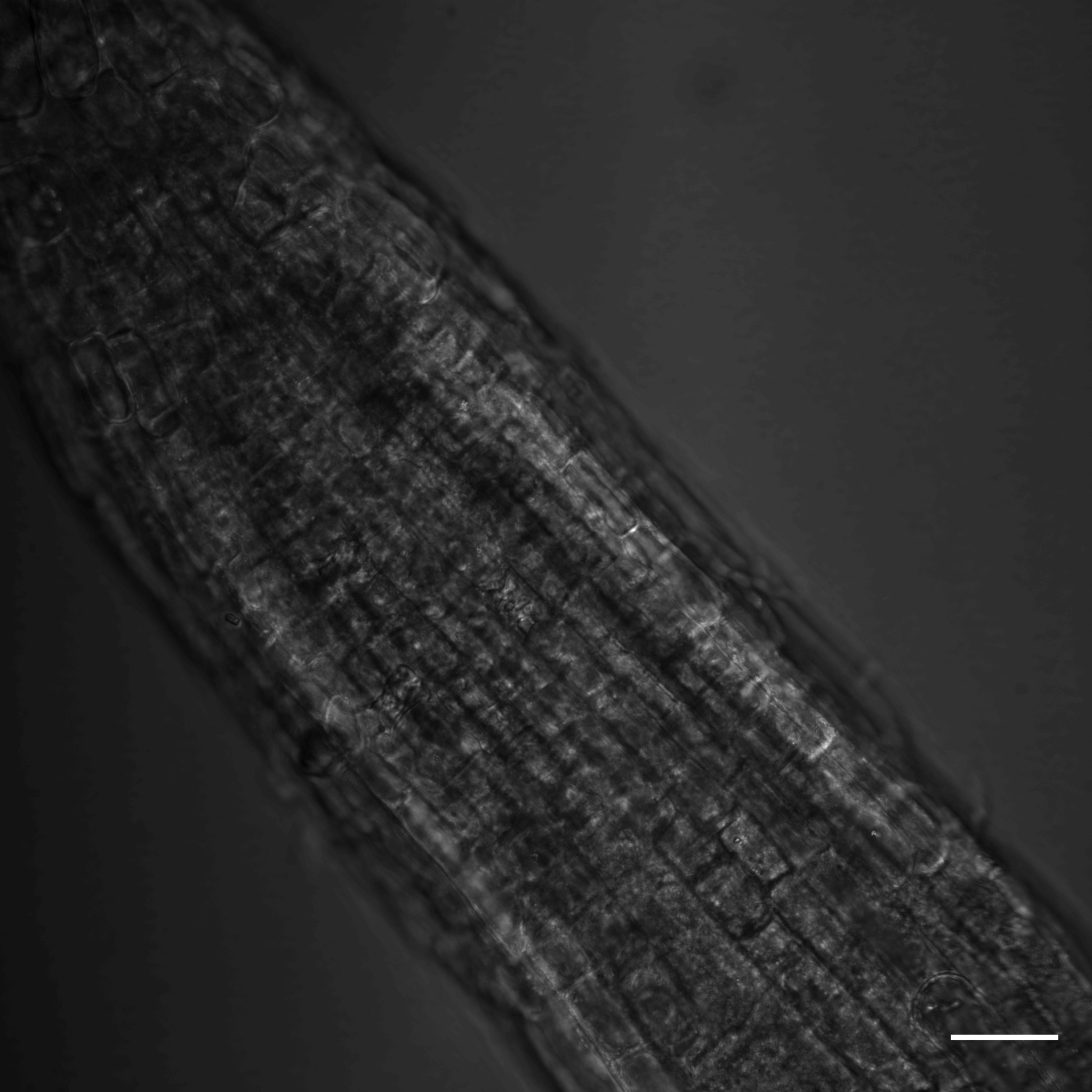

Supplement: Supplementary file 5 — Source data Fig. 4 [file 44319_2025_433_MOESM5_ESM.zip › Fig 4/4D/NT/BES1-YFP NT rt/Image 17_Maximum intensity projection_c2.tif]

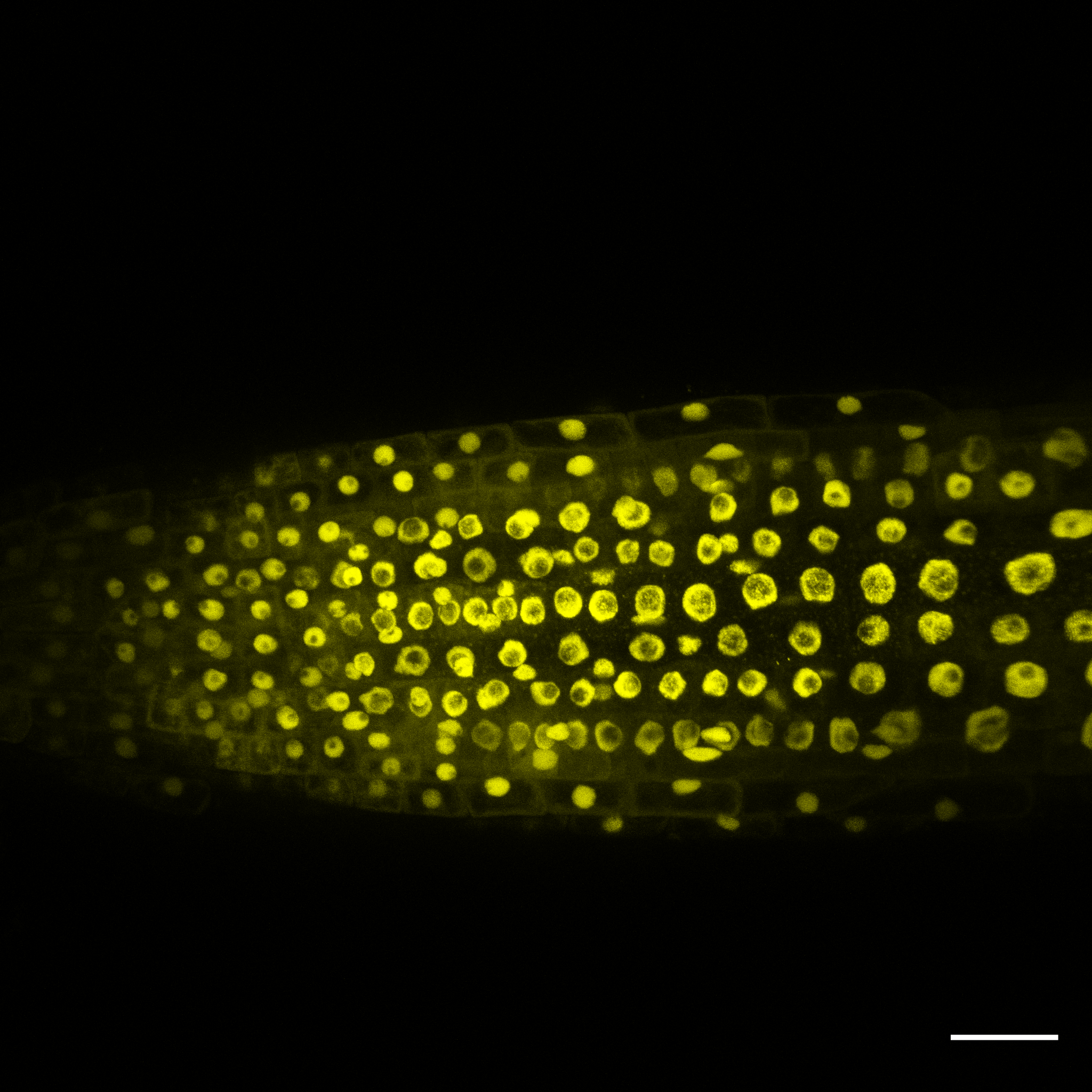

Supplement: Supplementary file 5 — Source data Fig. 4 [file 44319_2025_433_MOESM5_ESM.zip › Fig 4/4D/NT/BZR1-YFP NT rt/bzr ms RT Image 14_Maximum intensity projection_c1.tif]

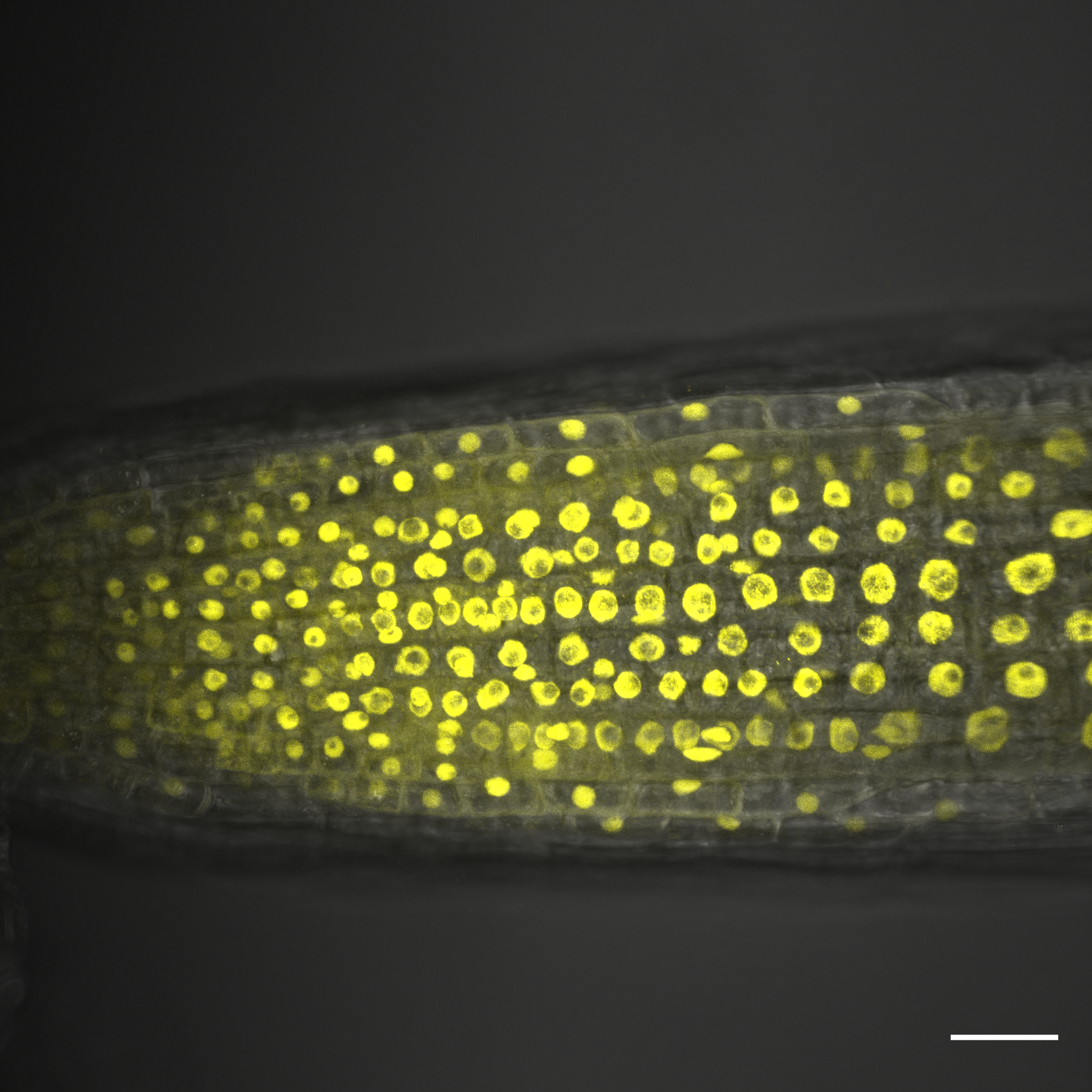

Supplement: Supplementary file 5 — Source data Fig. 4 [file 44319_2025_433_MOESM5_ESM.zip › Fig 4/4D/NT/BZR1-YFP NT rt/Image 14_Maximum intensity projection_c1-2.tif]

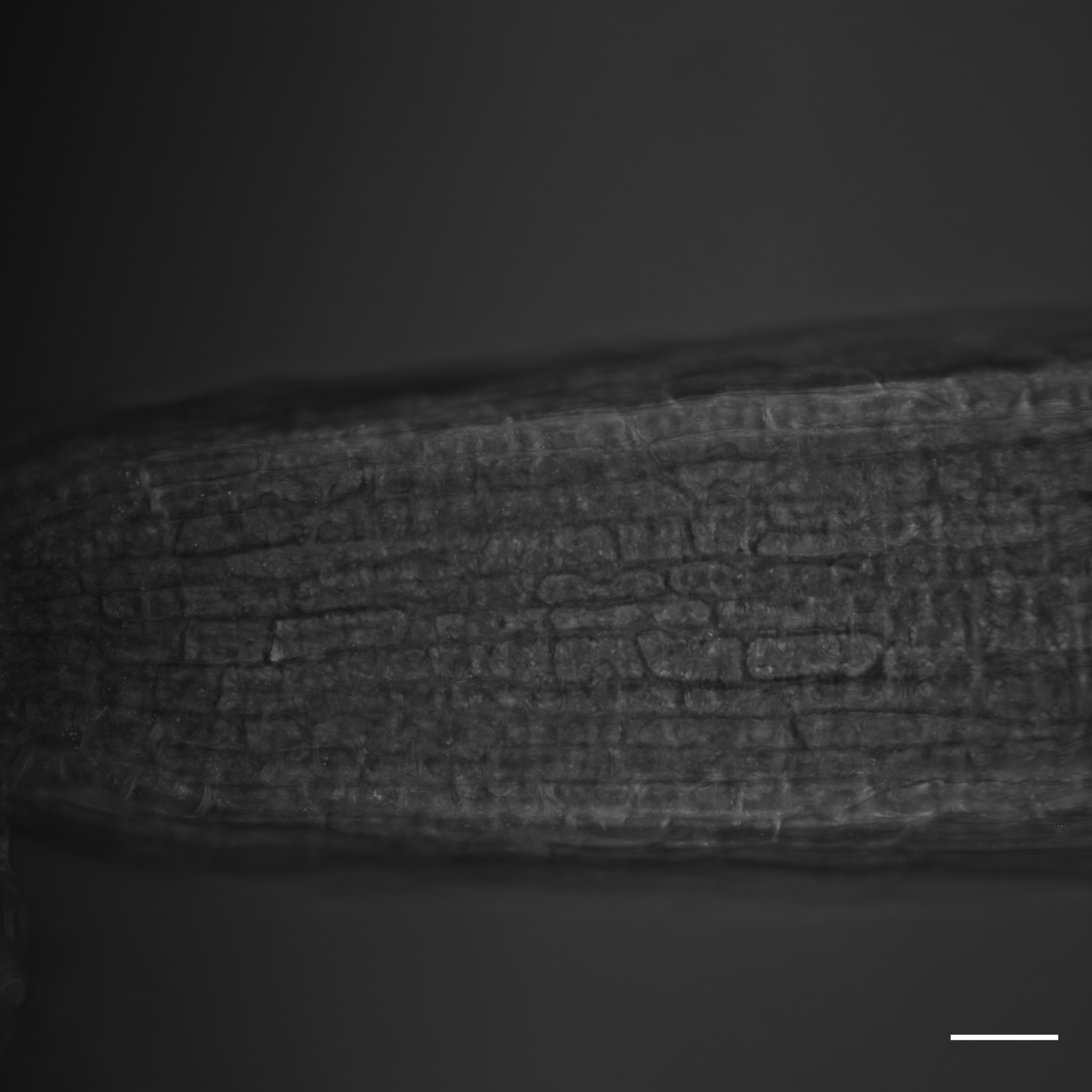

Supplement: Supplementary file 5 — Source data Fig. 4 [file 44319_2025_433_MOESM5_ESM.zip › Fig 4/4D/NT/BZR1-YFP NT rt/Image 14_Maximum intensity projection_c2.tif]

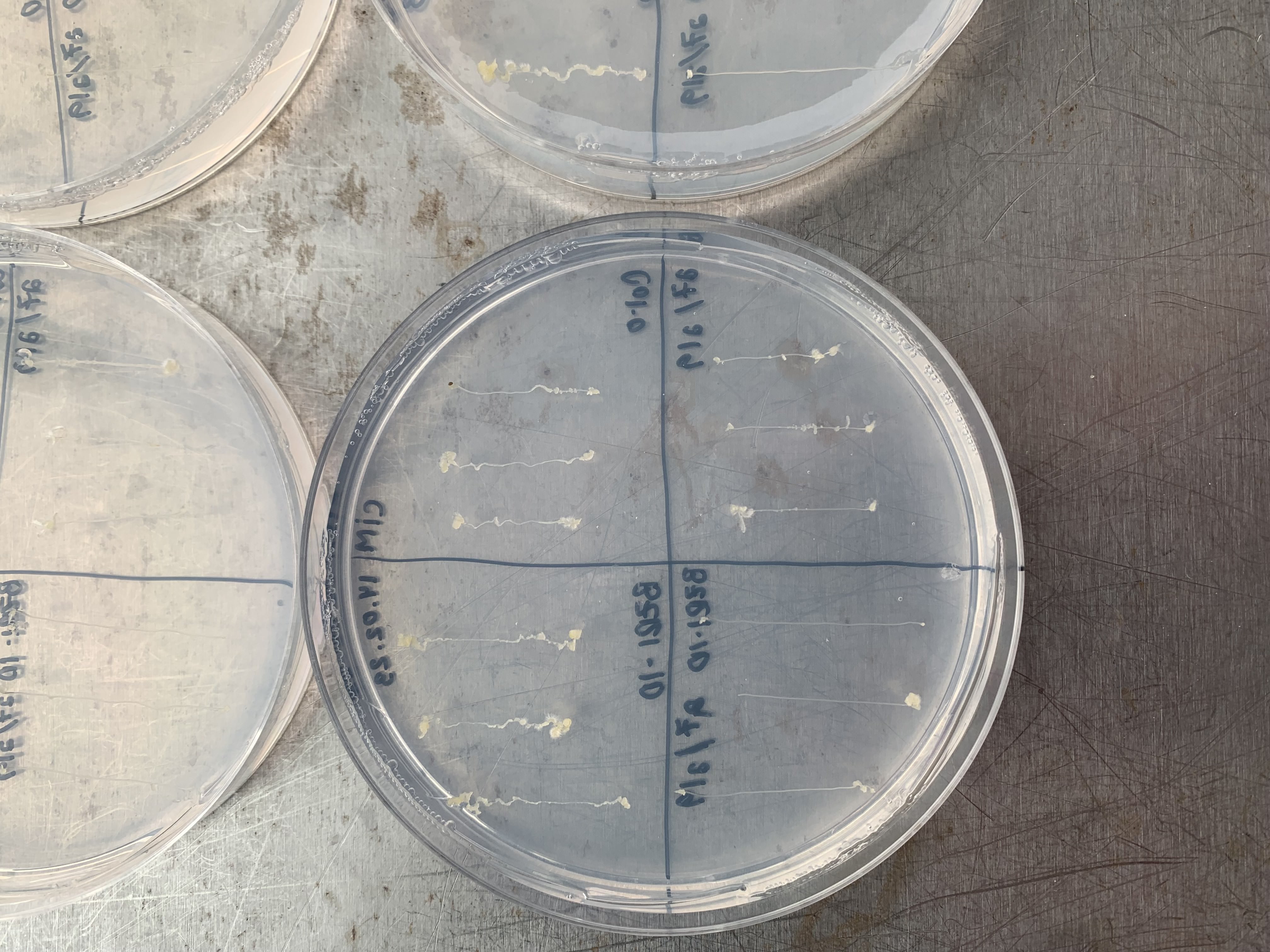

Supplement: Supplementary file 7 — Source data Fig. 6 [file 44319_2025_433_MOESM7_ESM.zip › Fig 6/6A/arf7-1-a19-2_IMG_2019.jpg]

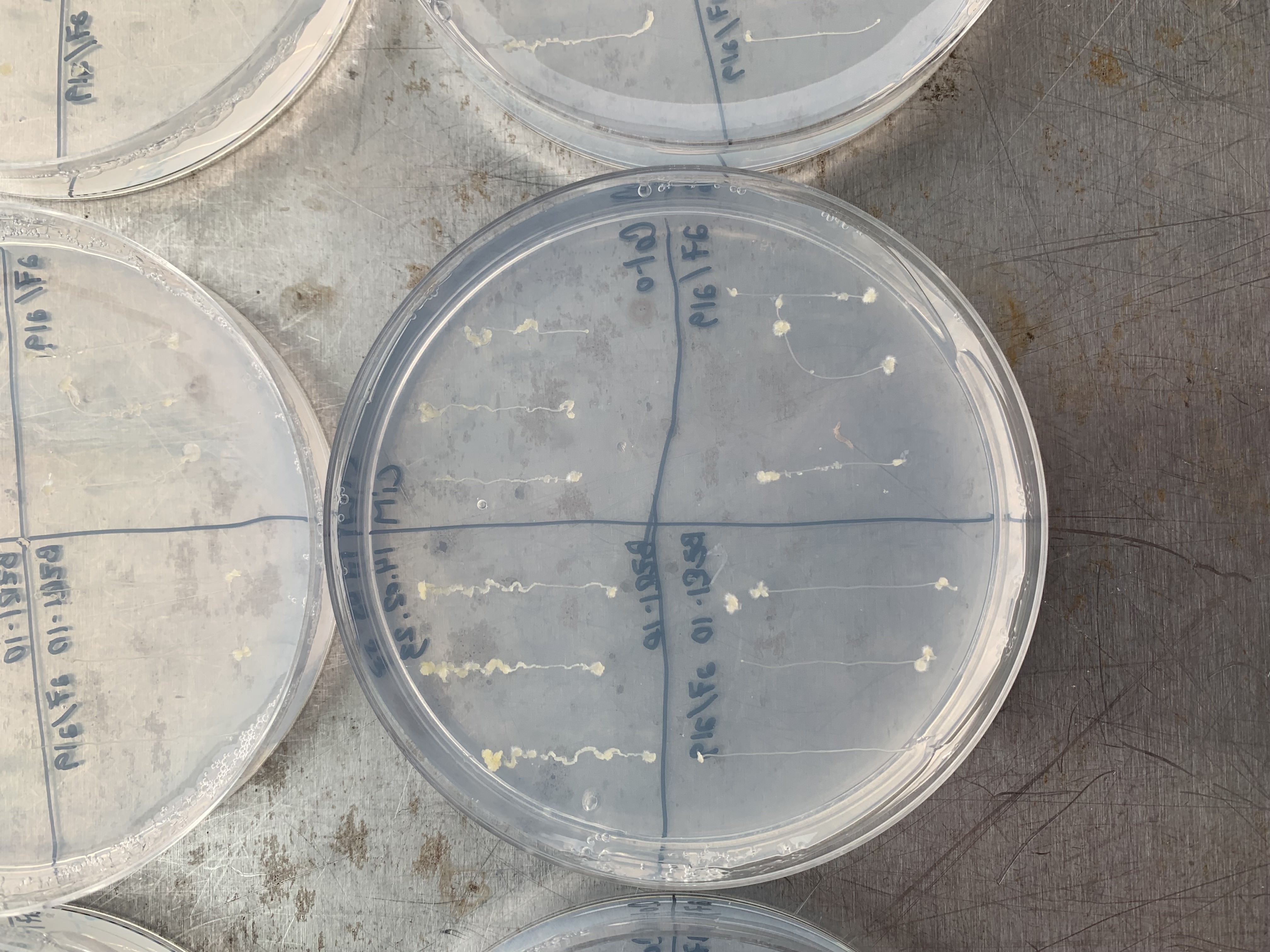

Supplement: Supplementary file 7 — Source data Fig. 6 [file 44319_2025_433_MOESM7_ESM.zip › Fig 6/6A/bzr1-D_IMG_2020.jpg]

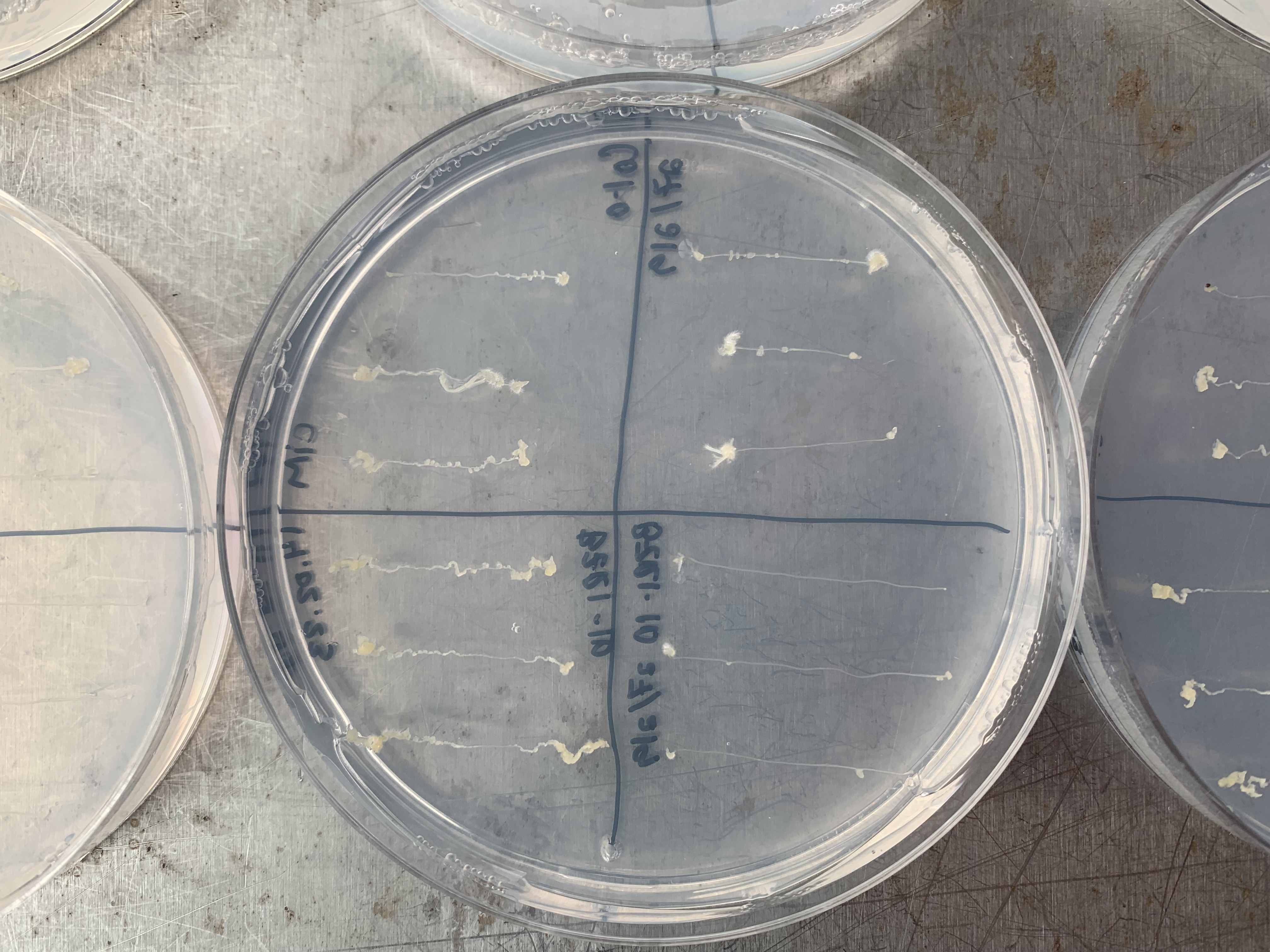

Supplement: Supplementary file 7 — Source data Fig. 6 [file 44319_2025_433_MOESM7_ESM.zip › Fig 6/6A/Col-0_bzr1-D-arf7-1-arf19-2_IMG_2022.jpg]

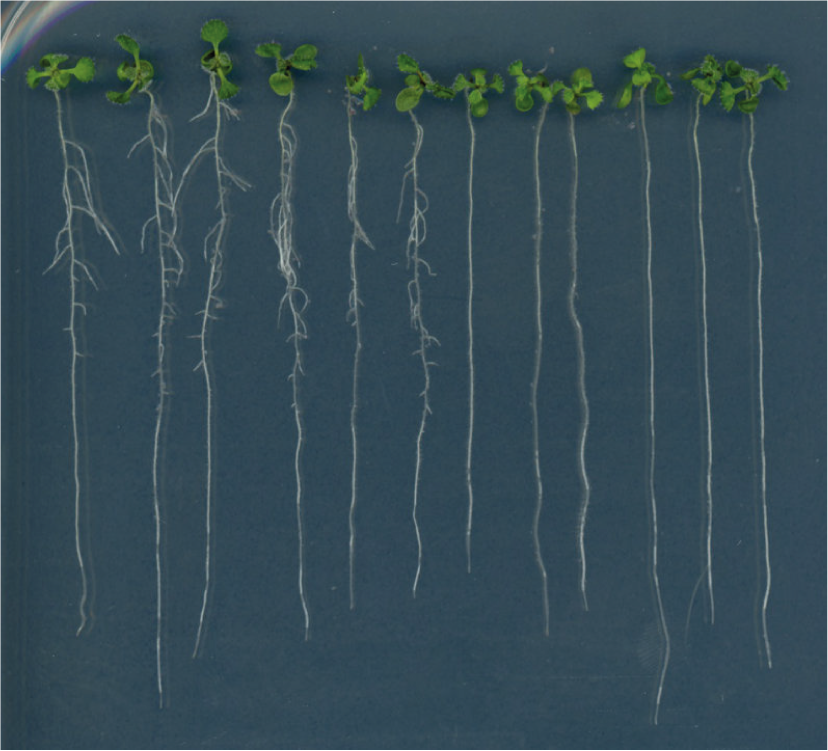

Supplement: Supplementary file 7 — Source data Fig. 6 [file 44319_2025_433_MOESM7_ESM.zip › Fig 6/6C/6C.tif]
