# Supplementary material for: BZR1 promotes pluripotency acquisition and callus development through direct regulation of ARF7 and ARF19
Source: EMBO Rep. 2025 Jun 26;26(14):3554–73. doi: 10.1038/s44319-025-00433-5 (PMC12287390; doi:10.1038/s44319-025-00433-5)
Supplement: Supplementary file 11 — Expanded View Figures [file 44319_2025_433_MOESM11_ESM.pdf]

Expanded View Figures

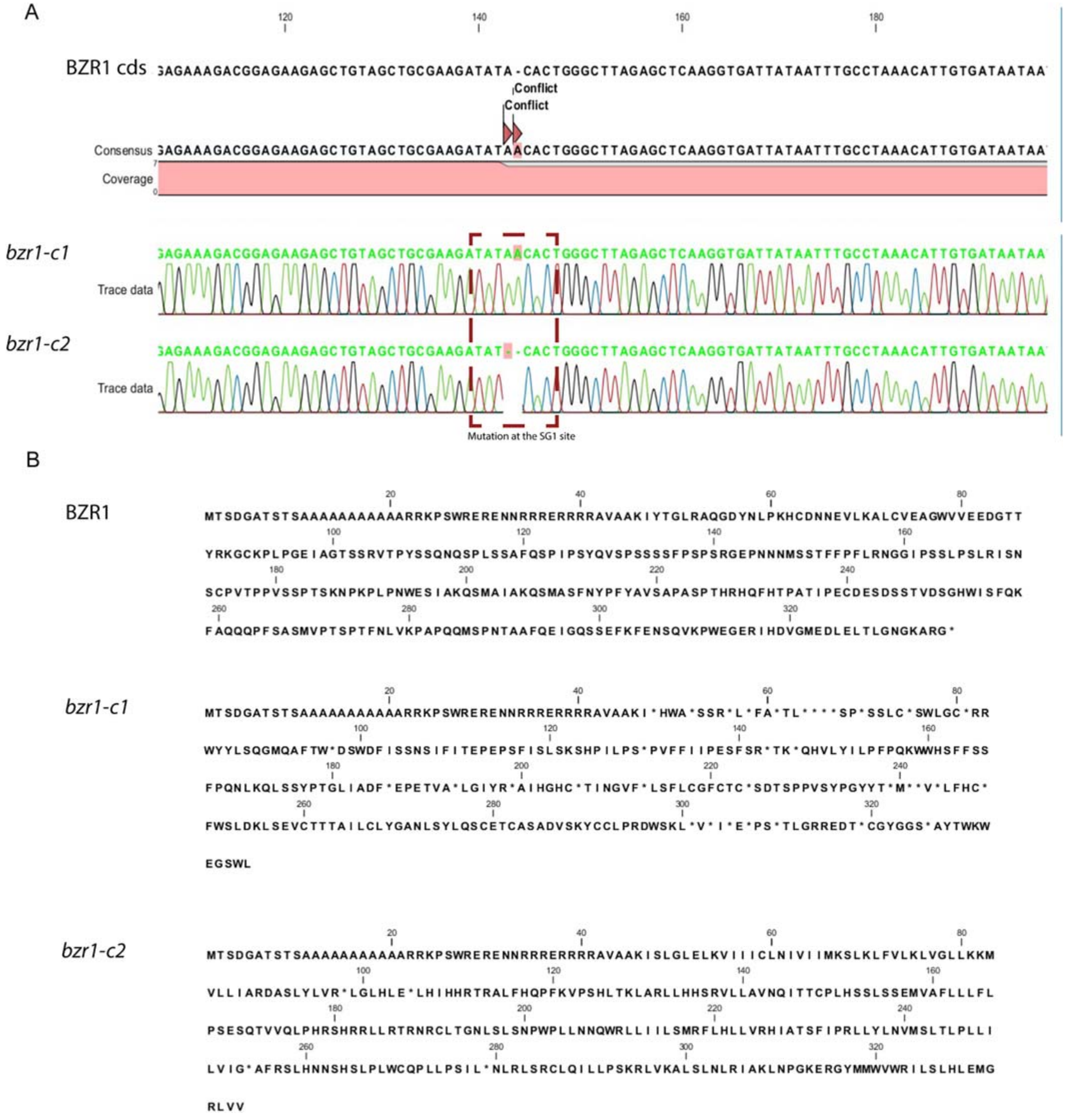

Figure EV1. Generation of *bzr1* CRISPR lines.

(**A**) *bzr1-c1* and *bzr1-c2* lines. DNA sequence for *BZR1* CDS and the two CRISPR–Cas9 generated BZR1 mutant lines *bzr1-c1* and *bzr1-c2* showing mutations to occur at position 144 and 143 (indicated by dashed square). (**B**) Protein sequence of translated BZR1 CDS. Frameshift mutation and premature stop codon are observed for *bzr1-c1* and *bzr1-c2*, respectively. See material and method for details.

 © The Author(s)

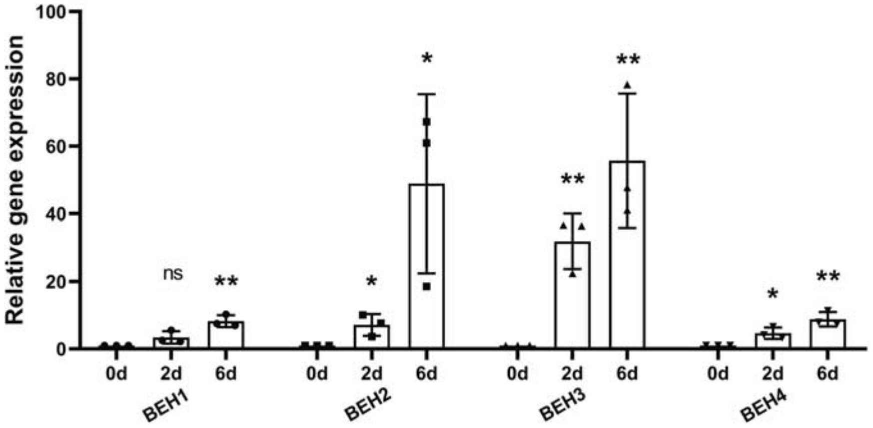

Figure EV2. *BEH1-4* is transcriptionally induced upon CIM treatment.

Rt-qPCR testing expression of *BEH1-4* after 2- and 6- days of CIM incubation, compared to 0 d treatment i.e. root explants. Tissue was collected from 7-day old seedlings and put in liquid CIM media for 2 or 6 days before collecting. Expression was normalized to *ACTIN2*. Bars display the mean and SD of $n = 3$ biological replicates each performed with 3 technical replicates. Statistical tests were made against 0 d. Statistical analysis was performed using unpaired Student's *t* test (*$P < 0.05$, **$P < 0.01$). *BEH1*: 0 d vs 2 d (ns $P = 0.087$), 0 d vs 6 d (**$P = 0.0020$). *BEH2*: 0 d vs 2 d (*$P = 0.0304$), 0 d vs 6 d (*$P = 0.0350$). *BEH3*: 0 d vs 2 d (**$P = 0.0030$), 0 d vs 6 d (**$P = 0.0087$). *BEH4*: 0 d vs 2 d (*$P = 0.0196$), 0 d vs 6 d (**$P = 0.0032$). Source data are available online for this figure.

© The Author(s)

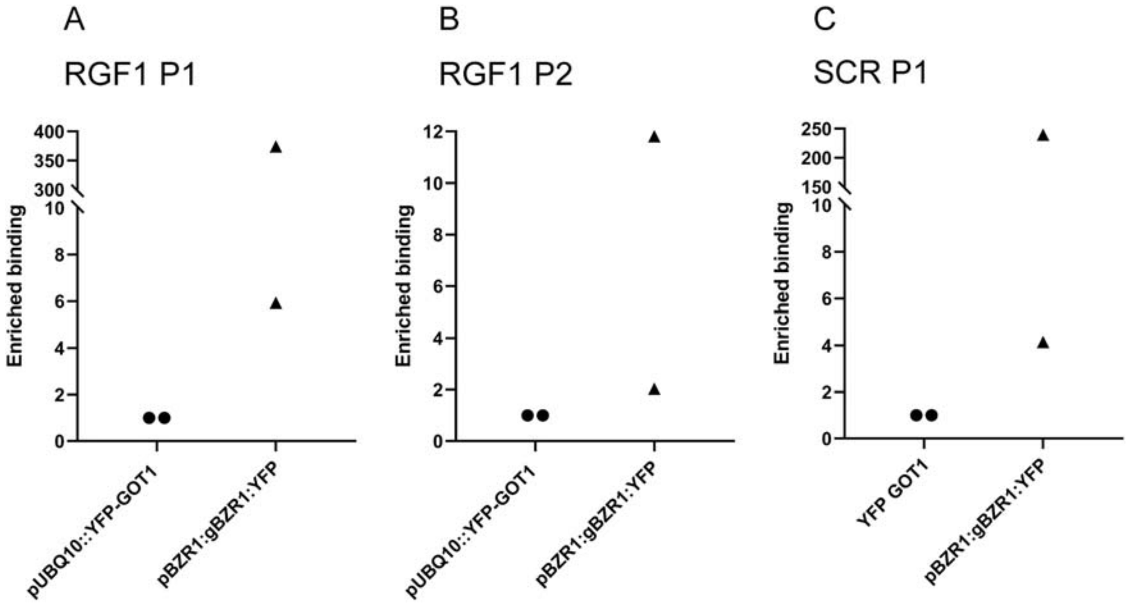

Figure EV3. BZR1-YFP bind to the promoter of *RGF1* and *SCR1*.

ChIP experiment showing the DNA binding of BZR1-YFP to RGF1 P1/P2 and *SCR1* promoter region. BZR1-YFP and YFP-GOT1 of callus explants after 21 days on CIM of *RGF1* P1 (**A**), *RGF1 P2* (**B**), *SCR1* (**C**). This was determined through quantitative RT-PCR. Fold change was calculated by normalizing to the YFP-GOT1 control for each sample. For each promoter region $n = 2$ biological replicates are shown. Source data are available online for this figure.

 © The Author(s)

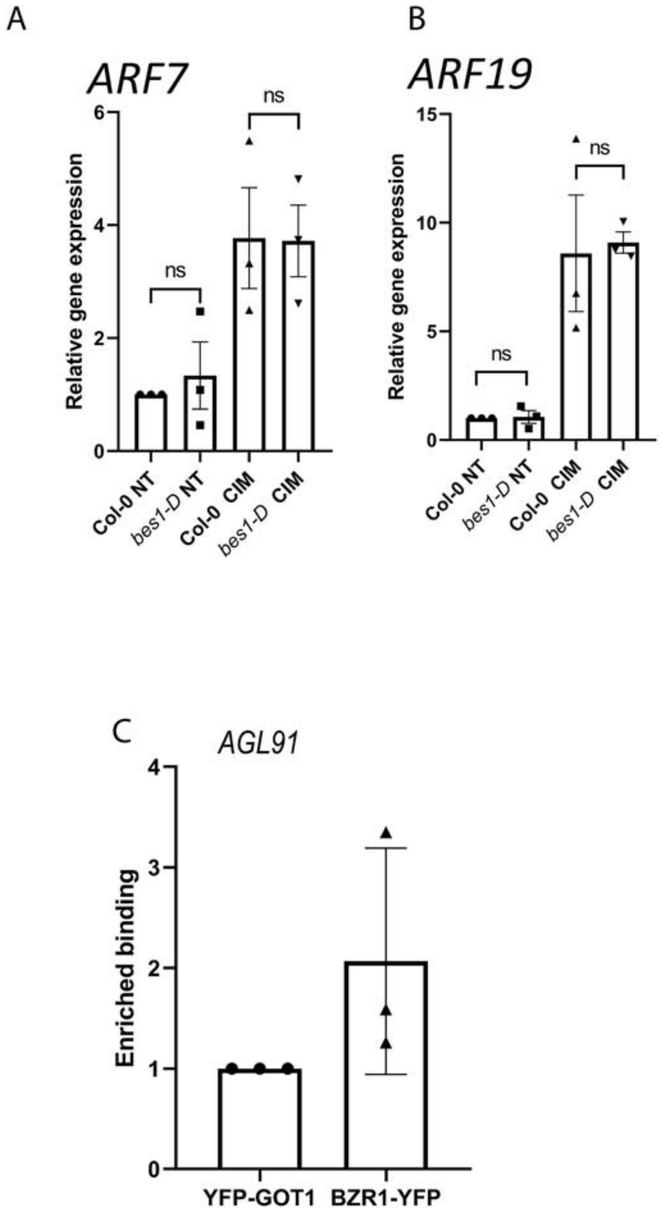

Figure EV4. Expression of *ARF7* and *ARF19* in *bes1-D* callus and ChIP-RT-PCR negative control.

(**A**, **B**) Relative expression of A) *ARF7* and B) *ARF19* were analyzed in Col-0 and *bes1-D*. Tissue was obtained from NT and 21-day CIM-treated root explants. Expression was normalized to *ACTIN2* and is relative to Col-0 NT; i.e. root explants from 7-day old seedlings grown on MS media. Bars display the mean and SEM of $n = 3$ biological replicates each performed with 3 technical replicates. Significance was calculated via student *t* test. Asterisks represent the significant difference from Col-0 NT/CIM from each treatment (*$P < 0.05$). (**A**) *bes1-D* NT vs Col-0 NT (ns $P = 0.60$), *bes1-D* CIM vs Col-0 CIM (ns $P = 0.9646$). (**B**) *bes1-D* NT vs Col-0 NT (ns $P = 0.8537$), *bes1-D* CIM vs Col-0 CIM (ns $P = 0.8644$). (**C**) Negative control of BZR1 ChIP-The DNA binding of BZR1-YFP to *AGL91* promoter region was determined through quantitative RT-PCR. Bars display the mean and SD of $n = 3$ biological replicates each performed with 3 technical replicates. Significance was calculated via Student's *t* test. BZR1-YFP vs YFP-GOT1 (ns $P = 0.175$), with ns representing a non-significant difference from YFP-GOT1 (control). Source data are available online for this figure.

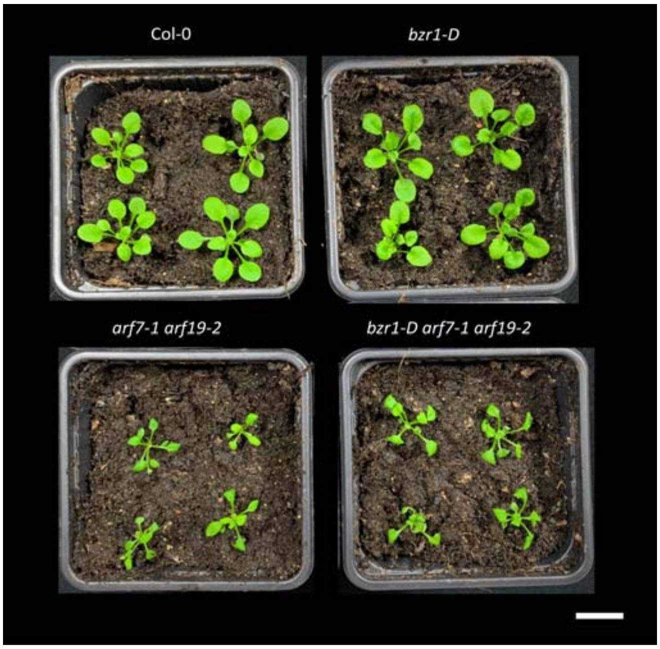

Figure EV5. Aboveground tissue of *bzr1-D/arf7-1/arf19-2* displays a similar phenotype as *arf7-1/arf19-2*.

Representative pictures of Col-0, *bzr1-D*, *arf7-1/arf19-2*, *bzr1-D/arf7-1/arf19-2* phenotypes of 2-week-old plants grown under standard conditions (See materials and methods). The observed phenotypes were consistent across multiple pots.

 © The Author(s)
